# Supplementary material for: Searching for proton transfer channels in respiratory complex I
Source: Biophys J. 2024 Aug 7;123(24):4233–44. doi: 10.1016/j.bpj.2024.07.041 (PMC11700350; doi:10.1016/j.bpj.2024.07.041)
Supplement: Document S1. Supporting materials and methods and Figures S0–S11 [file mmc1.pdf]

Biophysical Journal, Volume *123*

Supplemental information

Searching for proton transfer channels in respiratory complex I

Panyue Wang, Jackson Demaray, Stanislav Moroz, and Alexei A. Stuchebrukhov

Supplementary Information

Searching for Proton Transfer Channels in Respiratory Complex I

Panyue Wang, Jackson Demaray‡, Stanislav Moroz‡, and Alexei A. Stuchebrukhov*

Department of Chemistry, University of California at Davis, One Shields Avenue, Davis, California 95616

*Corresponding author: Alexei A. Stuchebrukhov,
Department of Chemistry, University of California at Davis.
E-mail: aastuchebrukhov@ucdavis.edu

‡These authors contributed equally.

0. Overall structure of the central axis water channel

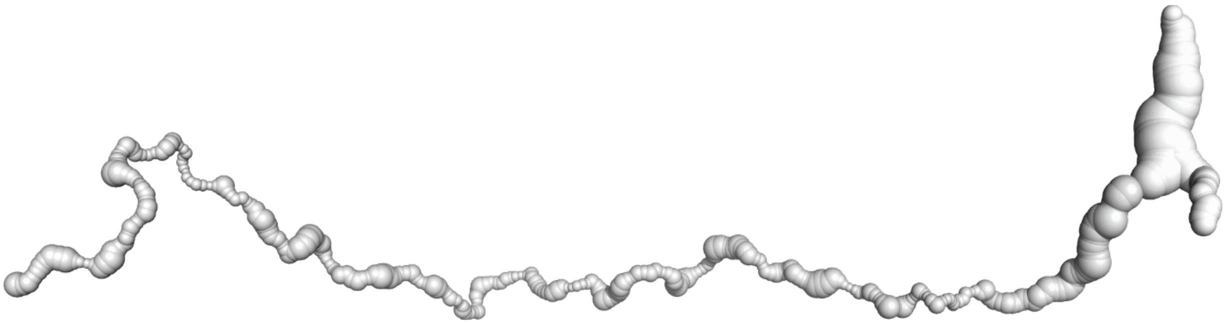

Figure S0. The proton transfer channel along the central axis with the quinone binding cavity shown on the right side of the figure. For better orientation, see Fig. 1 and 2 of the main text.

1. Central membrane part, subunits M and N

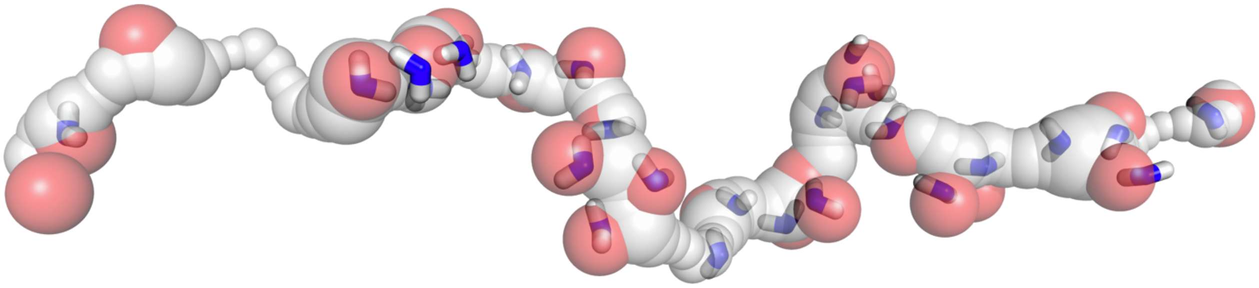

Figure S1. 26 experimental (red spheres) and 23 Dowser++ calculated (blue sticks) water molecules in the channel of *neutral* subunits M and N.

2. Left-most part (exit) of central axis, subunits L and M

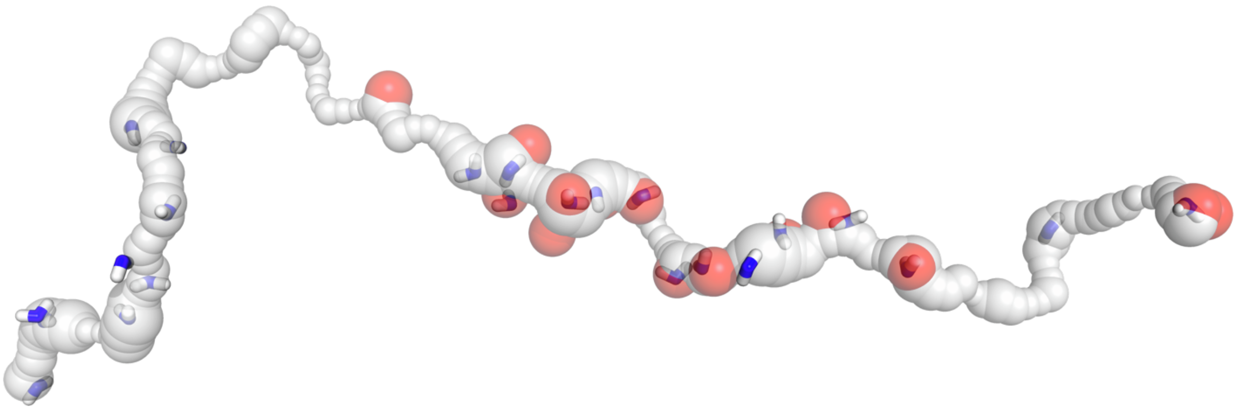

Figure S2. 14 experimental (red spheres) and 23 Dowser++ calculated (blue sticks) water molecules in the channel of *neutral* subunit L and M. For better orientation, see Fig. 1 and 2 of the main text.

3. The entrance of central axis: E-channel and JK interface. Detailed study of hydration

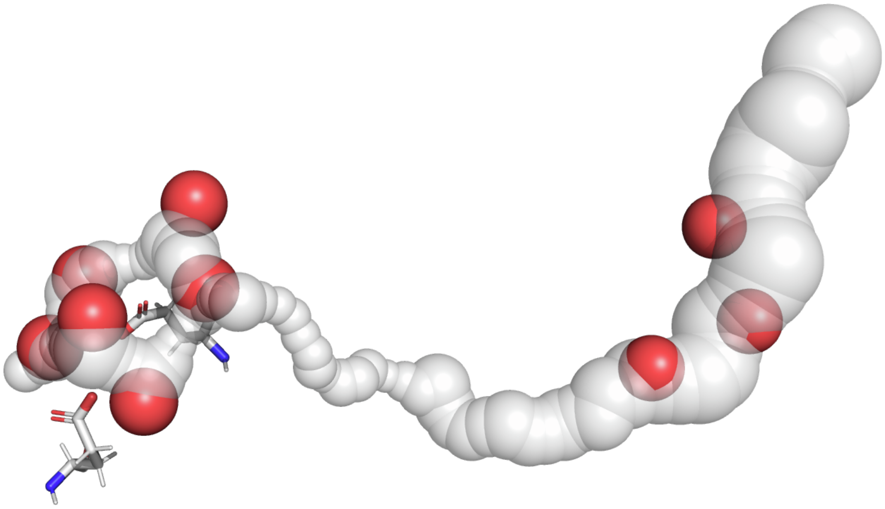

Figure S3. A noticeable difference in the structure of the E channel in two charged sates at JK interface (left-most part). Here shown is the channel structure that incorporates *both* charged and neutral states. 9 experiment water molecules are seen in the *combined* E channel. Two glutamic acids charged states result in a loop structure of the channel with experimentally resolved water molecules. This is an example of PT brunching structure of pathways/channels.

4. Comparing Dowser++ and MD energies of water insertion/removal

4.1. Using TIP3PP model of Dowser++, standard protonation states of the protein.

Shown in Figs. S4a and S4b below are three different ways to estimate binding energy of Dowser++ (D++) inserted water molecules.

A) Red - the original Dowser++ (D++) energy.

In D++, TIP3PP charges are scaled by factor 0.8 compared with TIP3P to reflect the less polar character of water in protein, TIP3PP, compared with the bulk model, TIP3P. After "draining", no interaction between water molecules are included, only interaction with the protein is counted. No relaxation of the inserted water molecules, nor the protein structure, is involved, except for rotational equilibration of hydrogen bonds [1-3].

B) Blue – Gromacs re-evaluation of the average potential energy of the inserted water molecules with TIP3PP model. This is the same water model as in D++ (TIP3P for Protein, TIP3PP model; charges are scaled by a factor 0.8). Both relaxation of water clusters and protein matrix are included. The average potential energy is evaluated along a 3ns dynamic trajectory of local structure.

C) Green - The binding energy is evaluated with Gromacs as in B), but now as a difference E(N)-E(N-1)_i of energies of two clusters with N and (N-1) water molecules upon removal of *i-th* water molecule from the cluster. The original cluster of N-molecules is formed by D++ procedure. The calculated energy value (with negative sign) corresponds to binding energy (work to remove a given water from the site), except that entropy is not counted. This energy is to be compared with the corresponding value in the bulk water -10kcal/mol. The remaining entropy change and the change in kinetic energy can amount to as much as 5kcal/mol. Thus, the actual cutoff energy is somewhere between -10 and 5 kcal/mol. The empirical threshold based on the analysis of experimental data of available structures is shown to be about -5kcal/mol.[1-3]

1. Morozenko, A.; Stuchebrukhov, A. A., Dowser++, a new method of hydrating protein structures. *Proteins* **2016,** *84* (10), 1347-57.

2. Morozenko, A.; Leontyev, I. V.; Stuchebrukhov, A. A., Dipole Moment and Binding Energy of Water in Proteins from Crystallographic Analysis. *J. Chem. Theory Comput.* **2014,** *10* (10), 4618-4623.

3. Farahvash, A.; Stuchebrukhov, A., Investigating the Many Roles of Internal Water in Cytochrome c Oxidase. *J. Phys. Chem. B* **2018,** *122* (31), 7625-7635.

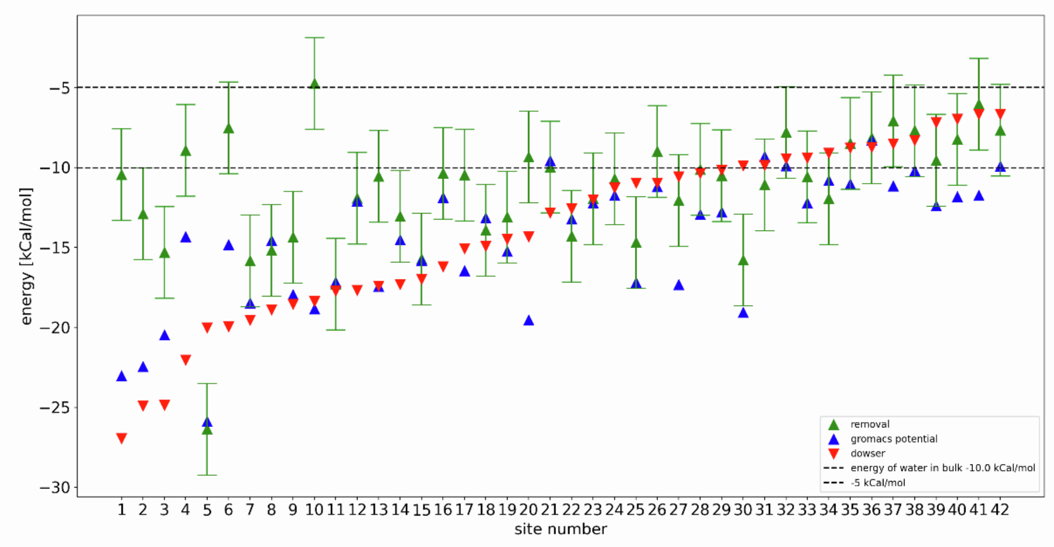

Figure S4a. Binding (removal) energy of Dowser++ water molecules in *charged* structure of extended E channel including JK subunits (see Figs. below). All Dowser++ inserted molecules were re-evaluated with Gromacs and TIP3PP model and remain below the threshold of -5kcal/mol.

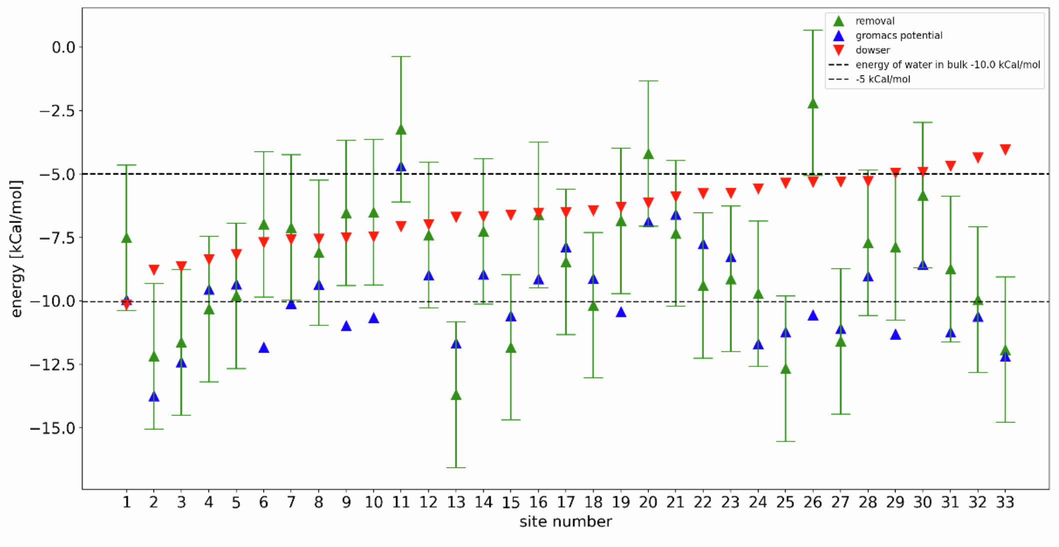

Figure S4b. Binding (removal) energy of Dowser++ water molecules in the *neutral* structure. Same as in Fig. 4a, but for neutral structure. Molecule i=11 is high in energy due to relaxation of the structure, 20 and 26 are due to HB clashes, removal of one stabilizes the other.

4.2. Effect of protein charge scaling.

Here we explored the effect of charge-scaling of protein charged residues, see Ref. [45] of the main text, Leontyev, I.; Stuchebrukhov, A., Accounting for electronic polarization in non-polarizable force fields. *Phys Chem Chem Phys* **2011,** *13* (7), 2613-2626. **Figs. S5-7** are informative as they give the scale of changes that occur upon the reduction (by a factor of ~0.7) of protein charges.

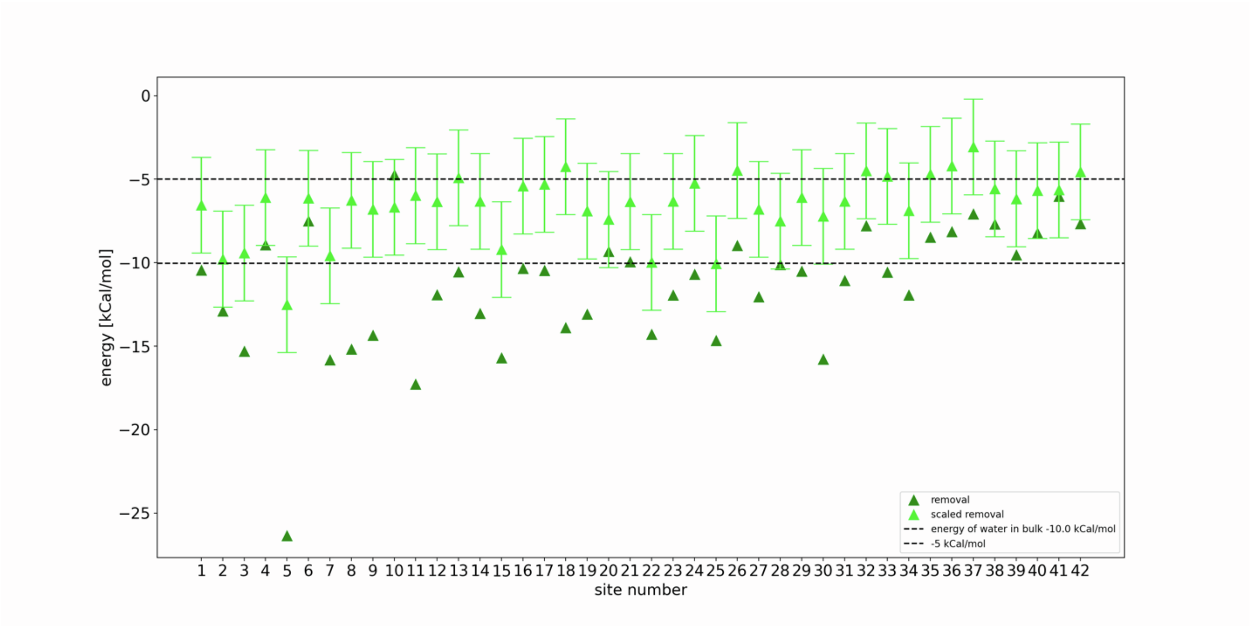

Figure S5. Binding (removal) energy of Dowser water molecules in *charged* structure of extended E channel including JK subunits. The charges of protonatable side chains are scaled (0.7) in calculations shown in light green. Almost all binding energies have increased except for water *i*=10 where scaled binding energy is ~2kcal/mol lower than unscaled calculations. This difference is within fluctuations shown as error bars in this graph.

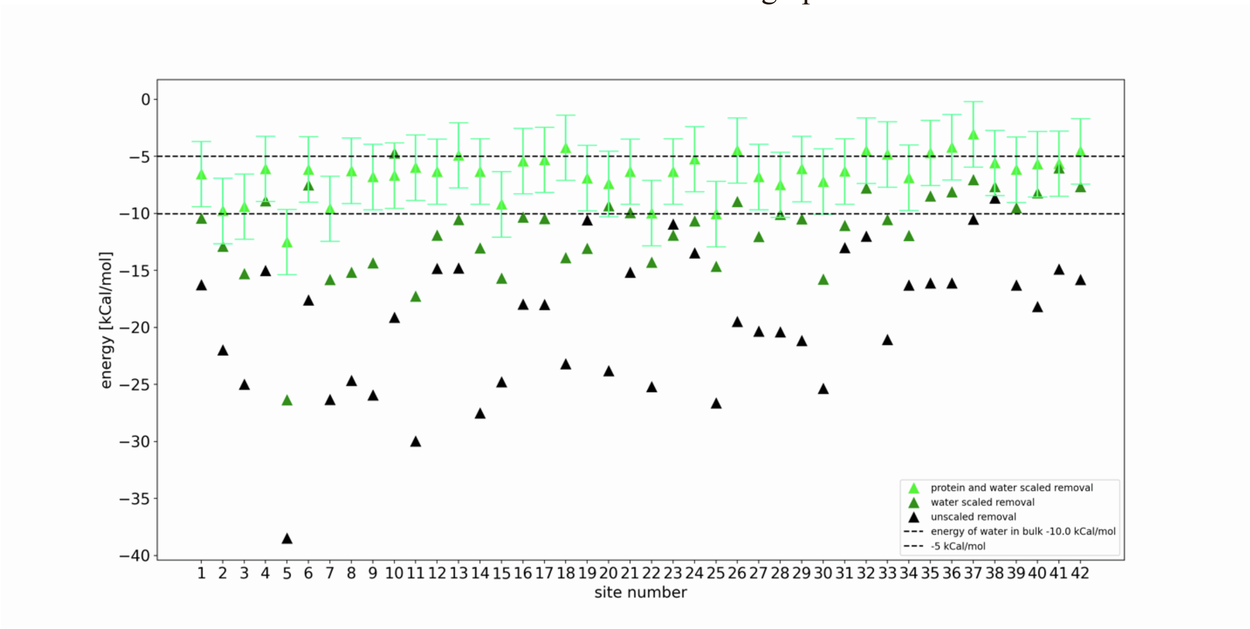

Figure S6. Binding (removal) energy of Dowser water molecules in *charged* structure of extended E channel including JK subunits. Both charges of protonatable side chains and charges of water are scaled in calculations shown in light green. The charges of water are scaled (TIP3PP) in calculations shown in dark green, while the charges of the protonatable side chains remain unscaled. For calculations shown in black, the charges of protonatable side chains and water are unscaled, which is the default setting of the CHARMM36 forcefield.

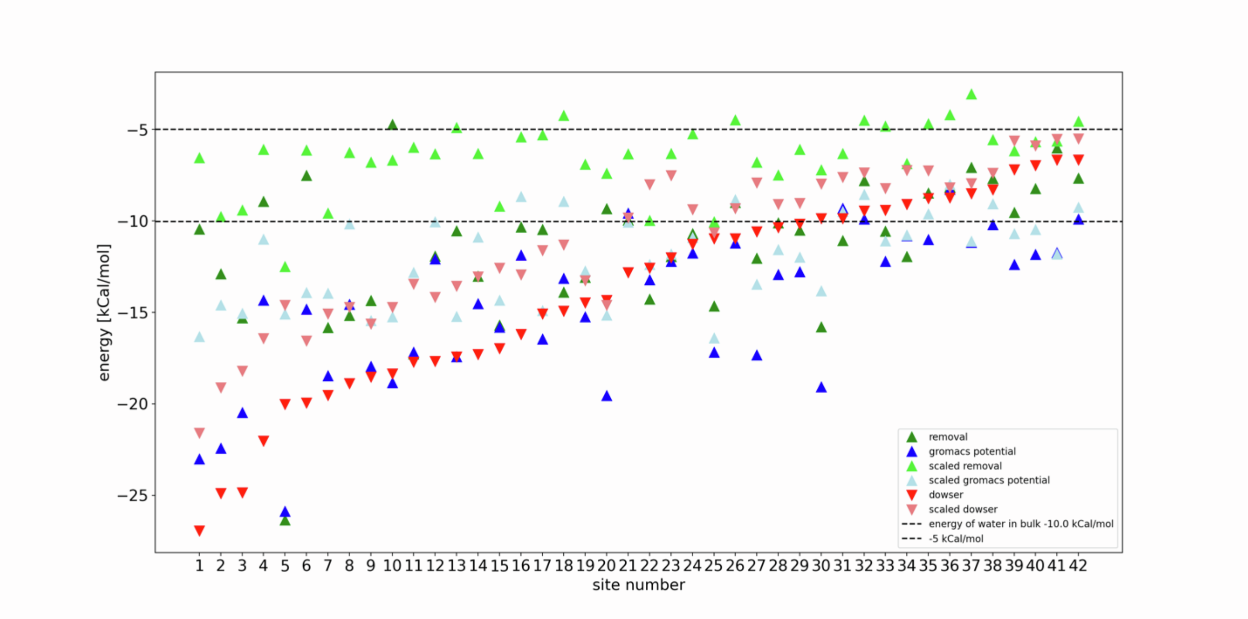

Figure S7. Summary of binding energy of water molecules in *charged* structure of extended E channel (including JK subunits) calculated by all 6 different schemes. The charges of protonatable side chains are scaled in calculations shown in light green, light blue and coral. The absolute energies of all schemes are rather different, but all still mostly below the likely threshold of -5kcal/mol.

5. E-channel hydration. Different hydration models predictions

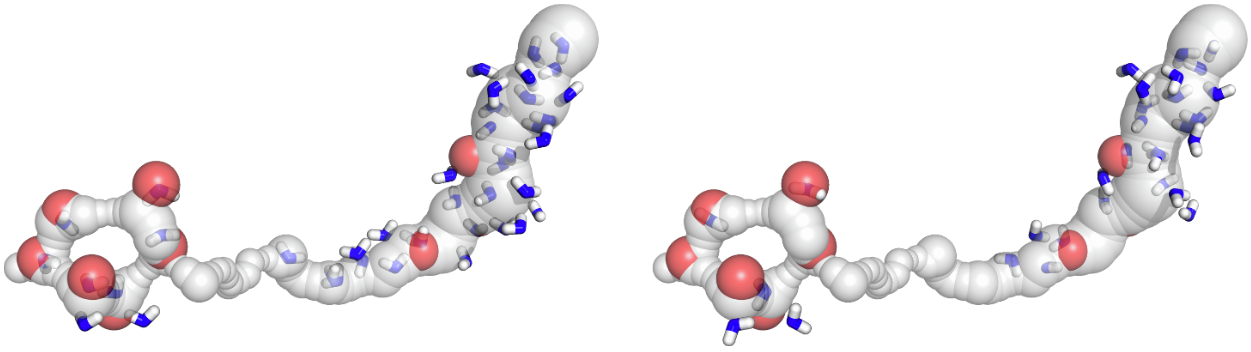

Figure S8a. 9 experimental (red spheres) and 41 Dowser++ calculated (blue sticks) water molecules in the charged (left) extended E-channel of subunits J, K, A and H vs. 30 Dowser++ calculated water molecules in the neutral (right) E-channel with removal energies lower than -5 kcal/mol. Notice, predicted water in the charged state of JK part of the channel (leftmost part) better matches experimental position of water molecules. Whereas predictions in the proper E-

channel (rightmost part, after the gap in water chain) apparently better match experiment in the neutral state, as the charged state grossly exceeds the observed 3 experimental water molecules for the whole range of possible cutoffs (5-10 kcal/mol) shown in Figs. S4-S7.

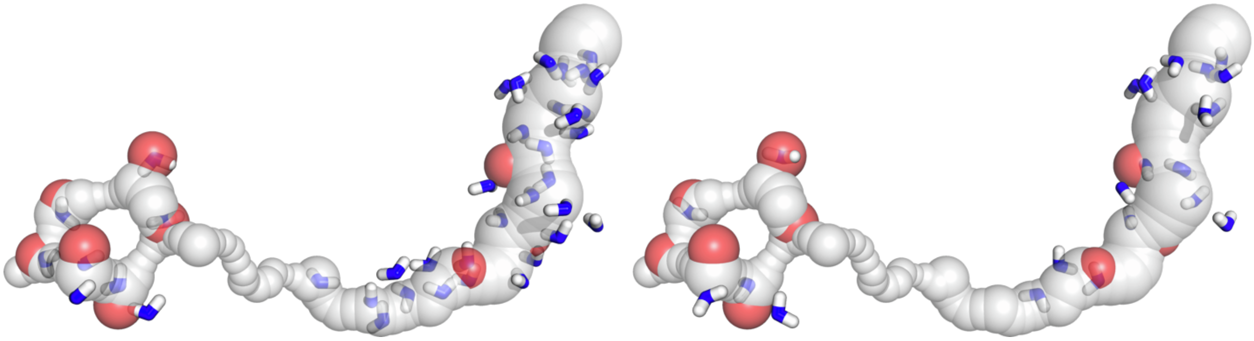

Figure S8b. 9 experimental (red spheres) and 39 Dowser++ calculated (blue sticks) water molecules in the charged (left) E-channel of subunits J, K, N, A and H vs. 20 Dowser++ calculated water molecules in the neutral (right) E-channel with removal energies lower than -7.5 kcal/mol.

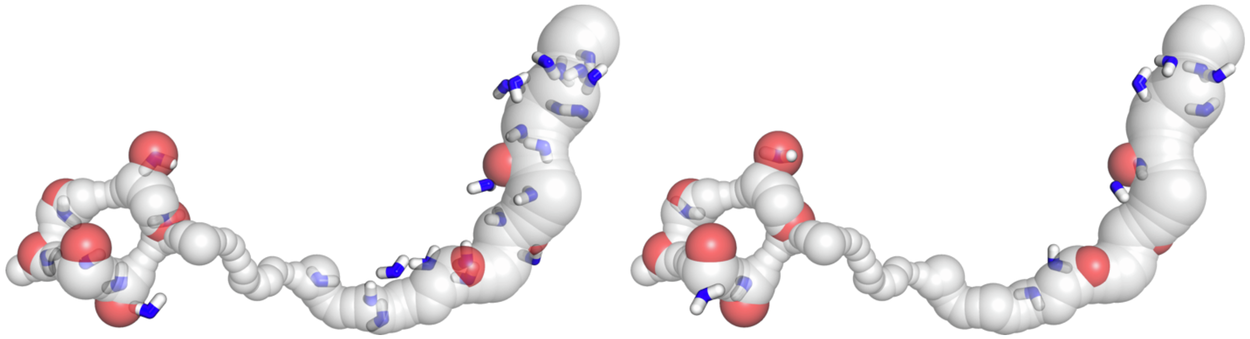

Figure S8c. 9 experimental (red spheres) and 27 Dowser++ calculated (blue sticks) water molecules in the charged (left) E-channel of subunits J, K, N, A and H vs. 9 Dowser++ calculated water molecules in the neutral (right) E-channel with removal energies lower than -10 kcal/mol.

Overall, our simulations indicate that the actual number of hydrated water molecules in the E-channel significantly exceeds those few that were observed in the cryo-EM structure of *Y. lipolytica* 7o71[20] but in line with, e.g., data for mouse complex I (see Ref. [17] in the main text, Grba, D. N.; Chung, I.; Bridges, H. R.; Agip, A. A.; Hirst, J., Investigation of hydrated channels and proton pathways in a high-resolution cryo-EM structure of mammalian complex I. *Sci Adv* **2023,** *9* (31), 1359). Similarly, more water is predicted for the central axis channel in L/ND5 subunit, as shown in the text. The reason for that could be poor resolution, or dynamic nature of molecules, or partial drying out of the samples during preparation; in particular, the latter seems likely in the E-channel connected to a big Q-cavity that could provide the channel of escape of hydrated water under drying conditions.

6. Insertion/Removal energies from protein water clusters (E-channel).

When there are clusters of hydrating water molecules, such as in the E-channel, to evaluate thermodynamic stability of the cluster the exact free energy of removal or insertion of individual water molecules needs to be calculated (and compared with the absolute chemical potential of bulk water). When one molecule is removed the whole cluster is affected due to water-water interaction within the cluster. Thus one needs to calculate the average energy of N and (N-1) water clusters and take their difference (removal or insertion energy). But this involves uncertainty due to thermal fluctuations of energies. Fig. S9 gives an estimate of energy distribution and uncertainty in such calculations.

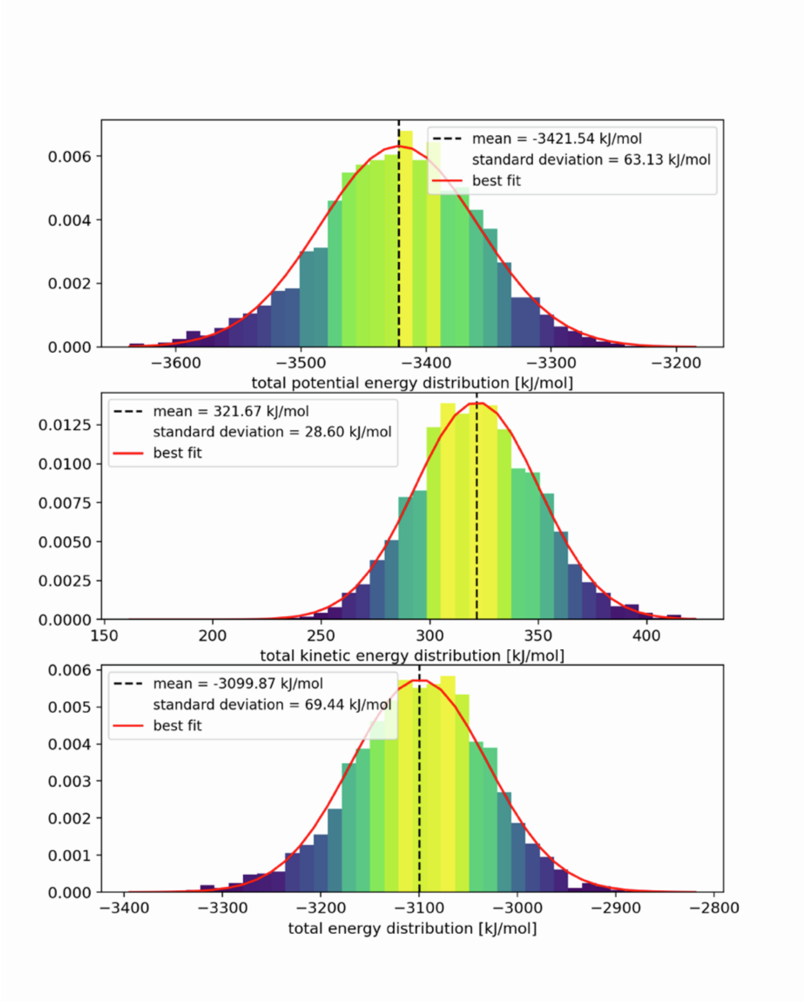

Figure S9. Energy (Potential, Kinetic, and Total) distribution of MD-simulated 42 Dowser++ water molecules in the *charged* structure of *Y. Lipolytica* extended E-channel. These and similar distributions were used to estimate the uncertainty (error bars) of the average energy of the clusters and resulting binding energies.

7. Effect of mutation in the gap region of E-channel (TM3/J/ND6).

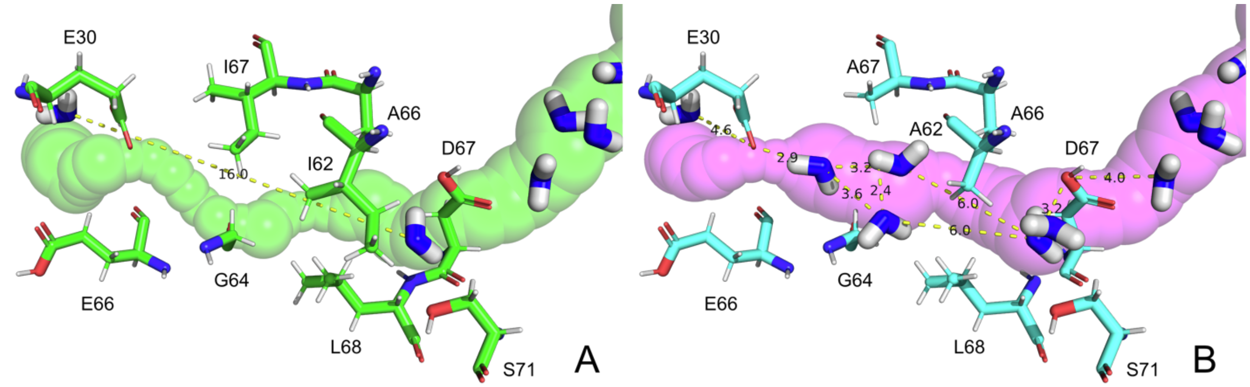

Figure S10. Shown in (A) is the channel gap between subunits A and K calculated using the *neutral* structure of *Y. lipolytica*. (The charged structure is shown in Fig. 12 in the main text.) The channel in (B) is calculated after mutating ILE 62 and ILE 67 into ALA 62 and 67. The Dowser++ predicted water molecules are shown as blue sticks. The nearby residues are shown as green sticks for the original structure and cyan sticks for the mutated structure. The 16.0 Å channel gap calculated for the neutral structure is shown in (A). Three additional water molecules were placed by Dowser++ due to the increased volume of the channel. These new water molecules broke the 16.0 Å gap into two smaller gaps of 4.6 Å and 6.0 Å, shown in (B). Notice that the same gap in charged structure, indicated by red arrows in Figure 12 (B), increased from 4.6 Å to 6.0 Å in neutral structure.

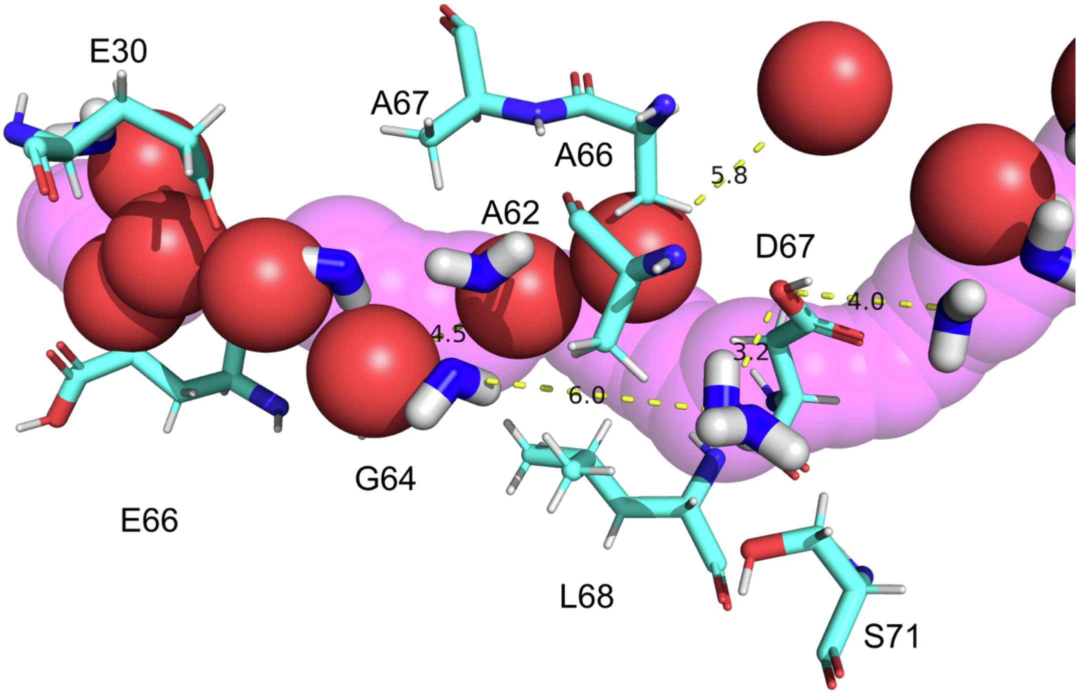

Figure S11. The channel between subunits A and K is calculated using the neutral structure of *Y. lipolytica*, after mutating ILE 62 and ILE 67 into ALA 62 and 67. Experimental waters from a newer mammalian structure [17] [1] (PDB code: 8OM1) are overlaid on top of *Y. lipolytica*

structure, shown as red spheres. The Dowser++ predicted water molecules are shown as blue sticks. The nearby residues are shown as cyan sticks. Three additional water molecules were placed by Dowser++ due to the increased volume of the channel. Two out of those three water molecules coincide with experimental waters. In addition, our prediction shows similar gaps of 6.0 and 4.0 Å long, comparing to 4.5 and 5.8 Å in the experiment. This 5.8 Å gap may be reduced by a nearby aspartic acid (D66 in 8OM1, D67 in 7O71 [2]) to 4.4 Å, as shown in [1].

[1] Grba, D.N., et al., *Investigation of hydrated channels and proton pathways in a high-resolution cryo-EM structure of mammalian complex I.* Sci Adv, 2023. **9**(31): p. eadi1359.
[2] Parey, K., et al., *High-resolution structure and dynamics of mitochondrial complex I-Insights into the proton pumping mechanism.* Sci Adv, 2021. **7**(46): p. eabj3221.
