# Supplementary material for: Searching for proton transfer channels in respiratory complex I
Source: Biophys J. 2024 Aug 7;123(24):4233–44. doi: 10.1016/j.bpj.2024.07.041 (PMC11700350; doi:10.1016/j.bpj.2024.07.041)
Supplement: Document S2. Article plus supporting material [file mmc2.pdf]

Biophysical Journal
Article

Biophysical Society

Searching for proton transfer channels in respiratory complex I

Panyue Wang,[1] Jackson Demaray,[1] Stanislav Moroz,[1] and Alexei A. Stuchebrukhov[1,*]
[1]Department of Chemistry, University of California at Davis, Davis, California

ABSTRACT We have explored a strategy to identify potential proton transfer channels using computational analysis of a protein structure based on Voronoi partitioning and applied it for the analysis of proton transfer pathways in redox-driven proton-pumping respiratory complex I. The analysis results in a network of connected voids/channels, which represent the dual structure of the protein; we then hydrated the identified channels using our water placement program Dowser++. Many theoretical water molecules found in the channels perfectly match the observed experimental water molecules in the structure; some other predicted water molecules have not been resolved in the experiments. The channels are of varying cross sections. Some channels are big enough to accommodate water molecules that are suitable to conduct protons; others are too narrow to hold water but require only minor conformational changes to accommodate proton transfer. We provide a preliminary analysis of the proton conductivity of the network channels, classifying the proton transfer channels as open, closed, and partially open, and discuss possible conformational changes that can modulate, i.e., open and close, the channels.

SIGNIFICANCE NADH: ubiquinone oxidoreductase, or complex I, is the first and largest enzyme in the electron transport chain. A massive network of putative proton transfer channels in the membrane part of *Y. lipolytica* complex I has been revealed by a computational analysis based on Voronoi partitioning of the protein structure. The network consists of the central axis proton transfer channel, beginning at the E channel of subunit H/ND1 and running through the membrane part of the enzyme, and side branches that connect the residues of the central axis to both sides of the membrane surface. Some proton transfer channels are big enough to accommodate water molecules that are suitable to conduct protons; others are too narrow to hold water but require only minor conformational changes to accommodate proton transfer. Many theoretical water molecules found in the proton transfer channels perfectly match the observed experimental water molecules in the structure; some predicted water molecules have not been resolved in the experiments. There are gaps in the proton conductivity of the central axis proton transfer channel, i.e., the channel is not uniform in conductivity but instead consists of several proton-connected patches, which are separated by gaps. We provide a preliminary analysis of the proton conductivity of the network channels, classifying the proton transfer channels as open, closed, and partially open, and discuss possible conformational changes that can open and close the channels.

INTRODUCTION

NADH: ubiquinone oxidoreductase, or respiratory complex I, is the first and the largest enzyme in the electron transport chain (ETC), which catalyzes the two-electron oxidation of NADH and the reduction of ubiquinone. The redox reaction, in which electrons are transferred over a chain of seven iron-sulfur (FeS) clusters, is coupled to the translocation of four protons across the mitochondrial or bacterial membrane (1–4). Together with other enzymes of the ETC, complex I pumps protons across the membrane and creates the proton gradient that drives the ATP synthase in the cell. The molecular mechanism of redox-driven proton pumping by complex I is key to understanding the bioenergetic system of the cell and its potential dysfunctions (5–7).

Recently solved structures of complex I (8–22) from both bacterial and mitochondrial sources have provided insights into how the enzyme might function (18–20,23–31); however, the exact proton-pumping mechanism of complex I remains unknown. This is mainly because the proton transfer pathways, often transient in nature, are not directly revealed in structural experiments and remain speculative. Computational analysis, therefore, is a necessary, if not

Submitted January 31, 2024, and accepted for publication July 30, 2024.
*Correspondence: aastuchebrukhov@ucdavis.edu
Jackson Demaray and Stanislav Moroz contributed equally to this work
Editor: Ana-Nicoleta Bondar.
https://doi.org/10.1016/j.bpj.2024.07.041
© 2024 The Author(s). Published by Elsevier Inc. on behalf of Biophysical Society.
This is an open access article under the CC BY license (http://creativecommons.org/licenses/by/4.0/).

crucial, step in resolving the mechanism of proton translocation by the enzyme, as the previous work on other enzymes of ET chain suggests (6). For proton pumping, the proton transfer channels need to be gated to prevent proton backflow; thus, the proton channels may be open or closed, i.e., have variable conductivity depending on the conformation of the protein, which adds complexity to the problem. This is on top of the complexity of complex I itself, which pumps four protons in addition to two protons consumed for the reduction of quinone.

In all organisms, the structure of the core part of the enzyme reveals an almost 30 Å tunnel-like void for ubiquinone binding (Q-chamber) that leads from the N-edge of the membrane up to the N2 FeS cluster (Fig. 1). The structure of the enzyme shows that the entrance to the Q-chamber forms a narrow bottleneck, which is conserved in all organisms (32,33). The simulation work has shown that the bottleneck presents a barrier for quinone's passage to the Q-chamber, indicating that conformational changes are involved in quinone binding to complex I. A squeeze-in mechanism has been proposed, where dynamic thermal fluctuations allow quinone to get in and out (33). (However, whether quinone gets out of the Q-chamber in the enzyme cycle remains debatable (34).)

Previous studies have also discussed the role of water in quinone binding; it can either stabilize ionic networks in the Q-chamber or reduce hydrophilic-hydrophobic interactions through water expulsion (28,35). Sazanov et al. have suggested a mechanism for complex I where water exchange between the Q-chamber and solvent assists quinone to enter and exit its binding site (see (28)). Individual water molecules have been detected experimentally inside the

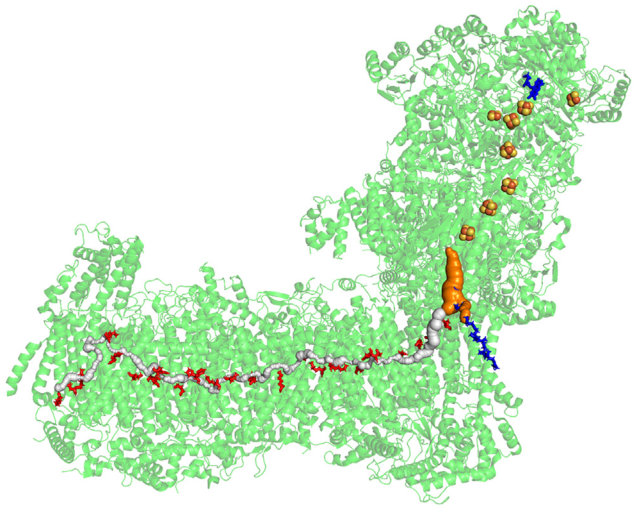

FIGURE 1 The revealed central axis putative proton transfer channel running through the membrane arm of *Y. lipolytica* (yeast) enzyme (20). The protonatable residues are shown as red sticks, which form the central axis. The quinone binding cavity is shown in orange on the right, with quinone sitting inside the cavity shown as blue sticks. Flavin mononucleotide is shown as blue sticks at the top, and the iron sulfur clusters (FeS) are presented as spheres.

Q-chamber complex I (16,17,19), though there is yet uncertainty about the extent of overall hydration in the Q-chamber in both Q-bound and apo forms (28,35).

Extending from the Q-cavity into the membrane arm of the enzyme, there is a chain of Lys and Glu residues that make up the so-called "central axis" along which the electrostatic/protonation signal propagates (20,25,26,36) in response to redox chemistry of quinone Q (Fig. 1). Recently, many water molecules have been resolved in the structure (16–22); however, how and where protons move (37,38) in the membrane part of the enzyme remain mostly unclear and speculative.

Here, we explore a strategy to identify the potential proton transfer channels using a computational analysis of a protein structure based on Voronoi partitioning, implemented in the popular program Caver (39), and applied it for the analysis of complex I from yeast *Y. lipolytica* enzyme (20) (Fig. 1). The analysis results in a network of connected voids/channels, which represent the dual structure of the protein; we then hydrate the identified channels using our water placement program Dowser++ (D++) and various other molecular dynamics (MD) techniques. Using this approach, a network of putative proton transfer channels in the membrane part is revealed. The network consists of the central axis proton transfer channel, beginning at the Q-cavity of subunit H/ND1 and running through the entire membrane part of the enzyme (Fig. 1), and side branches that connect the residues of the central axis to both sides of the membrane. The method provides a convenient rendering scheme of the potential proton channels, an alternative supplementing the usual continuous hydrogen-bonding analysis (17,40).

Many theoretical water molecules found in the channels perfectly match the observed experimental water molecules in the structure; some other predicted water molecules have not been resolved in the experiments. The channels are of varying cross sections. Some channels are big enough to accommodate water molecules that are suitable to conduct protons; others are too narrow to hold water but require only minor conformational changes to accommodate proton transfer. We provide a preliminary analysis of proton conductivity of the network channels, classifying the proton transfer channels as open, closed, and partially open, and discuss possible conformational changes that can modulate, i.e., open and close, the channels.

MATERIALS AND METHODS

The method to identify potential proton transfer channels is based on the idea that the proton transfer pathways consist of a series of protonatable protein groups connected by chains of water molecules (41,42). Water molecules, many of them (∼3000 in mt complex I but not all) resolved in current structures (16–22), are aimed to be predicted in the analysis in silico; those water molecules resolved in the structure are used for the validation of theoretical predictions. As water molecules require space inside the protein structure, we first search for the voids inside the structure that potentially can accommodate water molecules. Some voids should be connected to allow for water molecules to form connected chains along which protons

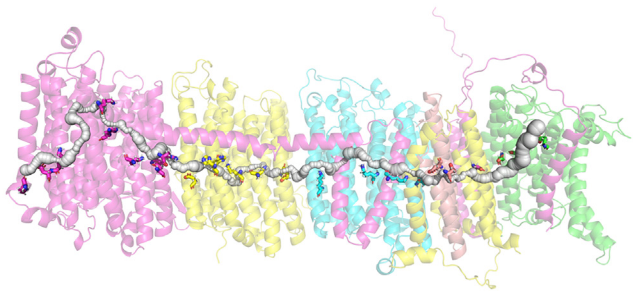

FIGURE 2 The putative proton transfer channel runs along the central axis in and between the core membrane subunits L, M, N, J, K, A, and H shown in different colors (in *E. coli* notation, *left* to *right*) of *Y. lipolytica*. Here and elsewhere, we use a convenient core subunit notation of *E. coli*. The charged residues are shown as sticks. Of particular interest are the inter-subunits regions, which appear to be connected (see also Fig. 13 and related discussion).

can be transferred. Our first step, therefore, is the analysis of a structure of the protein in search of connected voids within certain criteria, such as the minimum dimension of the void in a given location of the structure. One could naively assume a dimension of a sphere of radius 1.4 Å, which roughly corresponds to the size of a water molecule, but actually the criterion is more subtle (43), in particular in the view of searching for potential channels that may involve narrow bottlenecks and the effects of dynamics of the structure that may modify their conductivity. The narrow bottlenecks in the channel, blocking the free motion of water, can yet potentially be transparent for proton transfer with the assistance of protonatable groups (E, D, H, K, S, T, and Y) located nearby such bottlenecks. This can be included in the analysis of proton conductivity that follows.

To identify the empty space in the network of the protein atoms, we apply purely geometric analysis, assuming protein atoms to be rigid spheres of specific radii, and the empty space is defined as a sphere of maximum radius that can be inserted at a given location within the structure. For that, one can apply a well-known method of Voronoi partitioning, which selects manifolds of points (connected segments of lines in 3D space) equidistant from the nearest atoms of the protein. Obviously, a group of neighboring three atoms would provide the basis of a segment of a line that begins in the plane of three atoms, normal to that plane, and extends until a fourth atom would be found at the same distance. Only one point in 3D space would be typically equidistant from a general set of four other points. At this juncture, the segment of the line is joined by another segment that is defined by the new set of three atoms. Continuing this process, one can find a continuous path consisting of connected segments of straight-line intervals, each point of which is equidistant from the three neighboring atoms of the protein. This algorithm has been cleverly implemented in the computer program Caver (39) and, with some adaptation for our needs, was used in the first step of our analysis. (The program is well known in the field and was utilized before for structure analysis.)

The water channels have variable cross sections. Some channels are big enough to accommodate water molecules and suitable to conduct protons, while others have narrow bottlenecks that can be potentially overcome in proton transfer with the help of protonatable groups located nearby. With a reasonable criterion of minimal cross section radius, it is not clear a priori that such continuous channels should exist at all; yet, we find one such channel that runs through the entire membrane arm of the protein, over a distance of more than 200 Å (Fig. 1), which is quite remarkable by itself. Other channels will be described in the following text.

The next step involves water placement in the channels using our in-house software D++ (44,45), or some other water-insertion MD methods (see discussion in the supporting material), followed by the analysis of proton connectivity of the resulting paths. Here, we provide a preliminary analysis of the proton conductivity of the obtained network channels, classifying the proton transfer channels as open, closed, and partially open, and discuss possible conformational changes that can modulate, i.e., open and close, the channels. Most of the analysis was focused on the central axis, as it contains a lot of experimental water molecules for the validation of the method.

RESULTS

Central axis channel

Our first goal is to probe the putative proton transfer channel in the central part of the membrane arm of the enzyme that connects the key protonatable Lys and Glu(Asp) residues. Using our approach, we have discovered a continuous channel in the structure of (*Y. lipolytica*) complex I (see Figs. 1, 2, and S0). The channel starts from the Q-cavity (shown in Fig. 1 in *orange*), runs via the E-channel (28), and continues across the whole enzyme up to the leftmost L/ND5 subunit of the enzyme with the exit on the P-side of the membrane. Fig. 2 shows more details with subunits resolved and in different colors. What is remarkable is that the long-assumed continuous channel is indeed automatically found by the search algorithm and runs through the whole structure of some 200 Å long, connecting the central axis residues. Of particular interest are the inter-subunits regions, which appear to be connected (see also Fig. 13 and related discussion).

As shown in Fig. 3, the cross section of the channel varies along its length, which already indicates the nonuniformity in its possible proton transport properties that would be defined by hydrated water inside the channel, which we will address later. The cross section radius varies from 0.7 to 2.5 Å (Fig. 3), which indicates the whole range of water-binding properties—from likely binding to very unlikely. The data should be viewed keeping in mind the uncertainty of the structure (low resolution, B-factor in X-ray) and its possible thermal fluctuations. The quantitative analysis of the hydration of the channel is described in the following sections, which underscores the importance of regions with cross sections below 1.4 Å.

Side branches and half-channels

Using the same strategy, we next explored the side branches of the central axis channel that connect it to the upper (N-side) and lower (P-side) parts of the membrane. These branches make up the half-channels envisioned by Sazanov and colleagues (46) in their initial analysis of their structure. Fig. 4 shows all the side branches found in our analysis. It is seen that they are mostly rather narrow and not all hold bound water, as will be shown in the next sections. Generally, the network of all such interconnected voids/channels make up a dual (complimentary) structure alongside the usual protein structure.

In our strategy, we aim to identify all potential proton channels, whose conductivity properties will be specifically addressed later, one by one, keeping in mind that some channels should be closed and others open—for a given

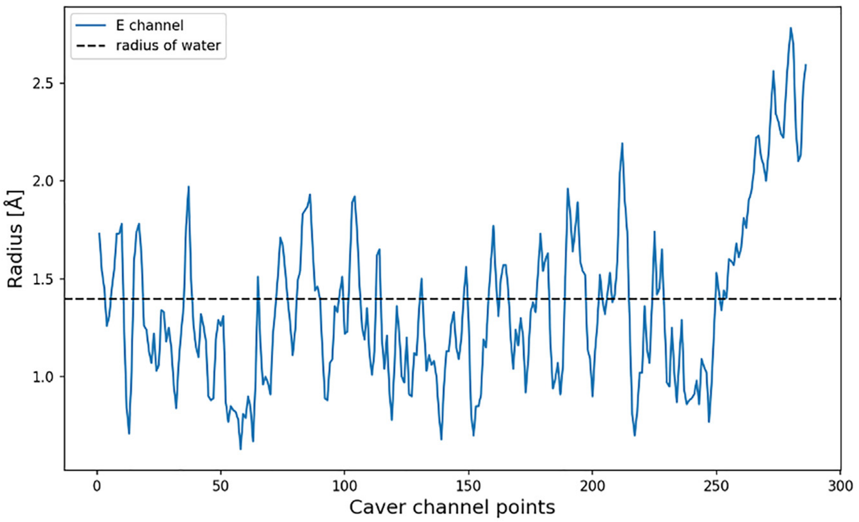

FIGURE 3 The cross section of the central axis channel, from subunit L to E channel, shown in Fig. 2. The radius of a water molecule, 1.4 Å, is shown as a black dotted line. The reference points at which the radius was measured are uniformly distributed along the length of the channel left (L/ND5) to right (E-channel), up to the connection to the Q-cavity.

snapshot of the structure; the conductivity may be expected to vary with slight structural changes that may occur along the turnover cycle of the enzyme or along the dynamic trajectory in the course of thermal fluctuations (30), as we have also seen in our recent study (47). It is crucial, however, to identify all potential channel candidates so that a directed study of conductivity properties of these candidates can be done in the next steps of the analysis.

Our theory is based on the assumption that for a given snapshot of structure, we can identify all possible candidate channels—open and closed. It is unlikely, but not impossible, that completely new channels would be formed or open for some unresolved or transient structures of the enzyme, as they require major structural conformational changes along the cycle. Again, it is not impossible or unknown (e.g., Rieske protein domain motion in complex III) for this to occur; however, it is unlikely in complex I, as no major structural changes have been seen in the membrane part so far (except for TM3/J/ND6 rotation, in the "open-close" transition (23,48,49), but even in this case, we can identify a connected channel, although of a narrow cross section, in the open state; see discussion below). Thus, we believe our strategy should reliably produce all potential proton channels that we can later classify as open or closed (not excluding permanently closed) and study their modulations with minor structural changes of the enzyme. Here, we should stress, however, that in the calculations of Fig. 4 (and Fig. 7 discussed later), we used only the core subunits of the enzyme; it is recognized that the supernumerary subunits and bound lipids will likely modify some of these side channels.

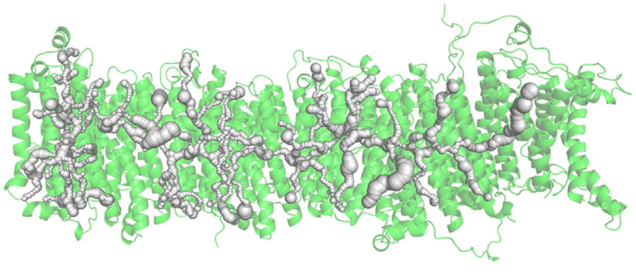

FIGURE 4 Vertical and horizontal side channels in the central axis of *Y. lipolytica* complex I membrane domain.

Water in proton transfer channels

In recent cryoelectron microscopy (cryo-EM) structures, many individual bound water molecules have been resolved (17,18,20), several hundred (~400–700) (17) only in the membrane part; it is understood that still not all water molecules are revealed in the experiments given their possible disordered nature at low temperatures. As our channels are defined by the empty space inside the protein structure, it is not surprising that many experimental water molecules are found in the predicted channels. Fig. 5 shows experimental water molecules of the central axis channel. However, not all parts of the channel are equally hydrated. For example, (in *Y. lipolytica* complex I (20)) the L/ND5 is visibly lacking resolved water, and there is an about 12 Å gap between the E-channel and the rest of the central axis channel (presumably in the so-called "open" state (49)), which visibly lacks the experimental hydration water.

With the understanding that not all water molecules are resolved in the structure, we use our water-prediction software D++ (44,45,50) to see where theoretical water molecules would be placed along the channel and how theoretical water molecules would correspond to the observed ones. Theoretical placement is based on the availability of empty space inside the structure and the energy of the bound water; the two parameters are connected, of course. In the first step, the energy is evaluated using combined semi-empirical methods of the D++ program, and in the latter, it is re-evaluated in (local) MD simulations using MD force fields. All

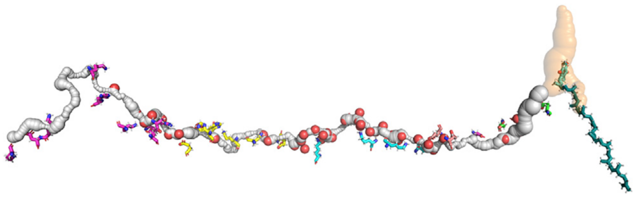

FIGURE 5 The central axis proton transfer channel with experimental water molecules (*red spheres*). Charged residues are shown as sticks. The quinone cavity, colored light orange, is shown on the right with quinone Q10 as sticks.

our MD simulations were performed using GROMACS software (51) with the CHARMM36 force field, including both the standard TIP3P water model and TIP3P modified for protein interactions, the TIP3PP model, with and without charge scaling of the charged protein residues (52), as detailed in the supporting material. This is all done to retain only water molecules with an energy below a certain threshold, which is a nontrivial subject of theory and typically taken around −5 kcal/mol (50) (see discussion in the supporting material).

The energy of a bound water molecule, among other things, depends on the charge state of the nearby residues (53). In our calculations, we assume the variable charge state (charged/uncharged) of the residues of the central axis (Lys and Glu), which is assigned manually, and all other residues are taken to be in the neutral state; this is done so as to efficiently take into account the action of counter ions and other charge-balancing factors in our computational procedures.

Since the actual charge state of the central axis residues in the structure is unknown (see discussion in (17,53)), running a theoretical prediction of water placement for different charge states and comparing them to experiments can provide a way to get insights into the charges of the residues of interest. (We should mention methods to predict pKa that we and others developed and have used before elsewhere (54–56) but which are not used here for the sake of keeping focus on the hydration issues, given that an accurate pKa calculation itself is not a trivial matter and is outside the scope of this paper.)

Fig. 6 shows predicted water molecules along the central axis channel together with experimental water for comparison. Here, all residues of the central axis are assumed to be charged.

A detailed analysis and discussion of specific regions of the channel will be given below. Here, we just mention that many predicted water molecules exactly match experimental ones, e.g., our predicted 27 molecules vs. 26 experimental molecules in the central part of the channel. The accurate comparison of predicted and experimental water molecules allows validation and further fine-tuning of the threshold energy in our water placement program D++ (see also discussion in the supporting material). With confidence gained in the regions of well-resolved water molecules, we predict the position of water molecules in the regions that lack experimentally resolved water molecules—in particular, in L/ND5 and M/ND4 subunits, as well as in the E-channel (see below).

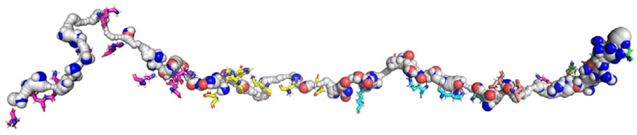

FIGURE 6 The central axis proton transfer channel with experimental (*red spheres*) and Dowser++-predicted water molecules (*blue spheres*). Charged residues are shown as sticks.

Using the method just described, we have extended our analysis to side branches of the central channel. The results are shown in Fig. 7. Both predicted and experimental water molecules are shown. Analysis and discussion of these results will follow in later sections of the paper.

Due to the complex nature of the system, we break it into relatively short segments and discuss specific details for each segment next.

Experimental and theoretical water molecules in the central axis channel

Here, we discuss the comparison of theoretical predictions and experimental water molecules in detail, focusing separately on the central part of the channel, comprising M and N subunits, the exit (left) part of the L and M subunits, and the entrance (right) part of the channel that connects the central axis channel to the Q-cavity via the E-channel (see Figs. 1 and 2). Experimental water molecules were obtained from cryo-EM structure 7o71 (20). Theoretical water molecules were calculated using our in-house software D++. The Caver channel points were used as docking focusing sites for D++. D++ uses both an empirical energy estimates by the Autodock Vina (57) algorithm and our built-in molecular algorithm. The energies of experimental and D++ water molecules were additionally re-evaluated by MD simulations (see additional discussion of related issues in the supporting material).

One of the ideas that we aimed to explore was comparing experimental water molecules and theoretical predictions for different charged states of the central axis residues and thereby possibly determining their actual charge state in the structure.

Central part of the channel M and N subunits

We ran D++ and MD calculations on the structures with different charge states. The structures were taken to be either fully charged (Lys+/Glu−) or fully neutral. We found that different charge states result in a slightly different channel geometry (due to influence of H-bonds), with corresponding variations of theoretical water positions. This effect is demonstrated in Fig. 8 (and Fig. S1): we have 27 theoretical

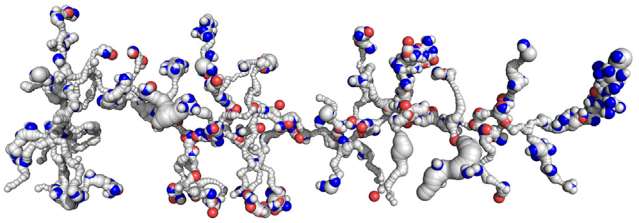

FIGURE 7 Water molecules calculated by Dowser++ (*blue and white spheres*) and found in experiment (*red spheres*) in the channel network of *Y. lipolytica* complex I membrane domain. For better orientation, see corresponding Figs. 2 and 4.

water molecules in the charged subunit M and N structure, but only 23 theoretical water molecules were found in the neutral structure, compared to 25 experimental ones.

However, here, the difference in comparison of theoretical predictions and experimental water molecules is too minor and does not allow for us to resolve with certainty the charged state of the central axis residues. This is due to apparently sufficient space to accommodate water molecules in either charge state; although the energies of hydrating water molecules are rather different, as expected, in both charged and neutral states, the energies are mostly below the threshold value and thus equally probable for hydration (see the supporting material for details), preventing discrimination of the two charge sates.

Fig. 8 *b* shows the comparison of D++ and GROMACS/TIP3P energies of the hydrated experimental water molecules. Naturally, after MD local structure minimization and optimization of hydrogen bonding, the GROMACS energies are mostly below semi-empirical D++ energies, except in some cases that involve local structure rearrangement in MD while, in D++ calculations, the structure is fixed and corresponds to protein models. The comparison is done for an additional validation of semi-empirical D++ energy estimates and thresholds.

Overall, given the quite remarkable agreement between theory and experiment, the data confirm the reliability of D++-predicted energies taken as a criterion of hydration (45). In particular, the analyses of some water molecules seen in experiment and of particularly high D++ energies (as W4 and W15 in Fig. 8 *b*) show that the cutoff energy of −5 to −6 kcal/mol assumed in D++ is a reliable criterion of hydration, i.e., we can trust theoretical predictions with D++ with energies below that cutoff (45,50). This means that in cases when theory does not match experiment, the likely reason is a low resolution of the experiment rather than an overestimation of theory. This will occur in the analyses of the next sections. An additional discussion of the insertion methods and corresponding simulation data are given in the supporting material.

Central axis L and M subunits

Fig. 9 shows the predicted and observed hydrating water molecules in the L and M subunits of the central axis channel. The data shown are for the charged state of the central axis residues; for comparison, the supporting material shows data for a neutral state. As shown in Fig. 9, D++ found many water molecules not seen in the experiment in the L subunit near the channel exit on the left (although many more molecules, but still not all, were found in other structures (17–19,21,22)). Also, it is worth noticing the potential gaps, lacking both experimental and theoretical water molecules, indicated by the arrows in Fig. 9 (see the related discussion in (17) and in the last section of this paper).

This end part of the central channel is of particular interest, as it was proposed to be the exit point of the channel by which all protons are expelled from the channel (28). Fig. 10 shows additional details of that region with predicted hydration water molecules together with the key Lys and Asp residues at the end of the channel.

Recent studies showed that a mutation of the lysine at the end of the channel had a significant impact on the proton translocation (7). This lysine is shown in Fig. 10.

E-channel connection to the central axis: The gap region (TM3/ND6/J)

Fig. 11 shows the rightmost part of the central channel that connects it to the Q-cavity via E-channel (see Figs. 1 and 2 for orientation). Only seven water molecules are resolved in the experiment in this part of the channel, while theory predicts much more significant hydration in both charged and neutral states of the channel. Additional data on energies in both charged and neutral states of E-channel are given in the supporting material. We have also explored different models of water and different criteria for threshold binding energy cutoffs, as described in the supporting material, to verify our predictions. Overall, our simulations indicate that the actual number of hydrated water molecules in the E-channel grossly exceeds those few that are currently observed in the cryo-EM structures. The same is true in the part of the central axis channel in the L/ND5 subunit, as shown earlier in this section. The reason for this could be poor resolution or partial drying out of the samples during preparation; in particular, the latter seems possible, as the E-channel is connected to a big Q-cavity that could provide the channel of escape of hydrated water under drying conditions. Overall, it is clear from various models that the E-channel is well connected for proton transfer, in agreement with previous theoretical analysis (40).

Of particular interest in Fig. 11 is the gap in water chain seen in the central part of the structure, which corresponds to the (TM3/J/ND6) region of the enzyme. Neither experimental nor theoretical placement predicts hydration water in this region (over ∼12 Å gap) in the explored structure 7o71 (20). Presumably, this is characteristic of only an "open" state, while in the "closed" state, the gap is hydrated (17,22). The current understanding is that conformational changes (rotation) of the TM3 helix of the J/ND6 subunit would allow for more space, and the connectivity of the

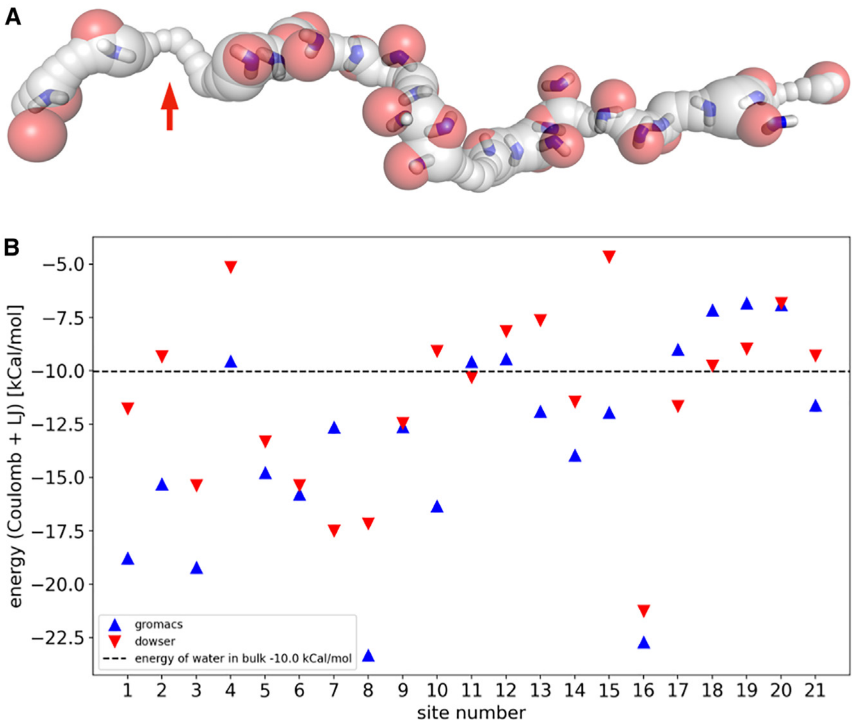

FIGURE 8 (*A*) 25 experimental (*red spheres*) and 27 Dowser++-calculated (*blue sticks*) water molecules in the channel of charged subunits M and N. Notice a potential gap in the channel indicated by the arrow (see discussion in the last section of the main text). (*B*) Verification of Dowser++ energy cutoff below −5 kcal/mol. Shown are energies of 25 experimental water molecules evaluated by GROMACS and (semi-empirical) Dowser++ in charged subunits M and N.

channel would be restored in the closed state (22,28,49). Presumably, the rotation of TM3 would result in the displacement of two isoleucines of the helix that would provide more space. This was explored in our simulations described in the next subsection. Additional data that are related to a difference in water placement in this part of the channel in two charge states are given in the supporting material, along with the detailed interface region between the gap and the entrance of the central axis channel at the K/N subunits.

Effect of mutation in the gap region of E-channel (TM3/J/ND6)

The bottleneck or gap between subunits A and K shown in Fig. 11 is created by two isoleucines, ILE 62 and ILE 67, of TM3/J/ND6 that block the channel (see Fig. 12 *A*). A rotation of TM3 upon the open-to-closed transition (that may or may not occur during the cycle (48)) was proposed (and seen in the experiment (22)) to result in the displacement of these residues, which would allow water molecules to penetrate this region and bridge the gap, making a connection of the E-channel to the central axis channel. To simulate the effect of the displacement during TM3 rotation, in which the isoleucines, in effect, are replaced by smaller residues that provide more space, here, we performed in silico mutations of the isoleucines to alanines and probed the effect of substitution on the formation of the channel (Figs. 12, S10, and S11).

Shown in Fig. 12 *A* is the 12.0 Å channel gap between subunits A and K calculated for the charged structure of *Y. lipolytica.* (The results for the neutral structure are given in Figs. S10 and S11.) The channel in Fig. 12 *B* is calculated after mutating ILE 62 and ILE 67 into ALA 62 and ALA 67. The radii of the gap (points 5–15) were significantly below 1.4 Å in the native structure, which increased to around 2.0 Å after the mutation. The D++-predicted water molecules are shown as blue sticks. The nearby residues are shown as green sticks for the native structure and as cyan sticks for the mutated structure. In the mutated structure (Fig. 12 *B*), two additional water molecules were placed by D++ due to the increased volume of the channel. These new water molecules reduced the E-channel gap from 12.0 to 4.6 Å, as indicated by the red arrows in Fig. 12 *B*. In the neutral structure, the remaining gap is increased to 6.0 Å, as shown in Fig. S10. Similar gaps are shown in Fig. 2 D of (17).

Fig. 12 (and also Figs. S10 and S11) shows that, indeed, the substitution of isoleucines to alanines can open the channel for hydration to accommodate an additional 2–3 water molecules. Despite a simplistic modeling of the results of conformational change, there is an almost exact match of our predicted molecules for *Y. lipolytica* and those observed in the mouse structure of (17) in the mutated region of the channel (see Fig. S11). However, it should not be overlooked that the additional hydration does not completely bridge the gap, and there remains a 4.6 Å break (for charged, and 6.0 Å for neutral structure) in the channel, as shown in Fig. 12. Thus, the question of proton conductivity here remains open. In our current estimate, the remaining gap should present at least a significant barrier for proton transfer, if not completely isolating the E-channel from the

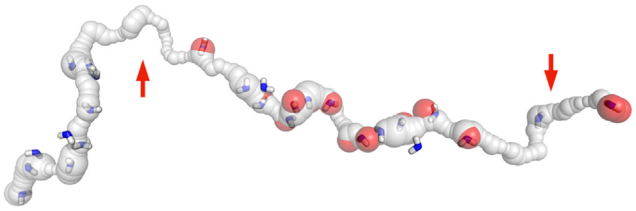

FIGURE 9 13 experimental (*red spheres*) and 27 Dowser++-calculated (*blue sticks*) water molecules in the channel of charged subunits L and M. Potential gaps, lacking both experimental and theoretical water molecules, are indicated by the arrows (see relevant discussion in the last section of the main text). For better orientation, see Fig. 2.

central axis. In further discussion, we will assume a possibility of a disconnected channel and explore its consequences.

Water in half-channels

We have done a similar analysis of the placement of water in vertical half-channels (although, admittedly, with much less detailed study at this stage)—the branches of the central channel that connect it to the upper (P-) and lower (N-) parts of the membrane surface. The results are presented in Fig. 7. With water placement in both central channel and half-channels, we can proceed to the discussion of possible circuitry of proton transport in the membrane part of the protein.

DISCUSSION

To discuss the proton circuitry of the system, we now need to address the proton conductivity of the identified channels. Whereas accurate quantification of proton conductivity is a challenging problem (58), which will be addressed elsewhere, here, the conductivity will be evaluated in a crude way: by separating conducting and nonconducting junctions by the distance between neighboring water molecules along the path (17,40) and looking at nearby protonatable residues that can help to carry protons over the gaps. Admittedly, such connectivity analysis is rather qualitative; however, we do not aim here to quantify the proton transfer within the apparently connected patches, but rather, our goal is to discriminate between the apparently connected and disconnected regions and to answer the key question of whether the central proton channel is continuous or not. The key result of the analysis is that there are clear gaps that are obviously not conductive for protons. The presence of isolating junctions along the central channel is of fundamental importance, as they discriminate between the two possible mechanisms of proton pumping (29). The conclusions are based on our prediction of water molecules in the channel (or rather the absence thereof in the gaps) for which we have confidence, as we validated our water insertion method in comparison with a large body of experimental data in the central part of the channel (see the supporting material for details).

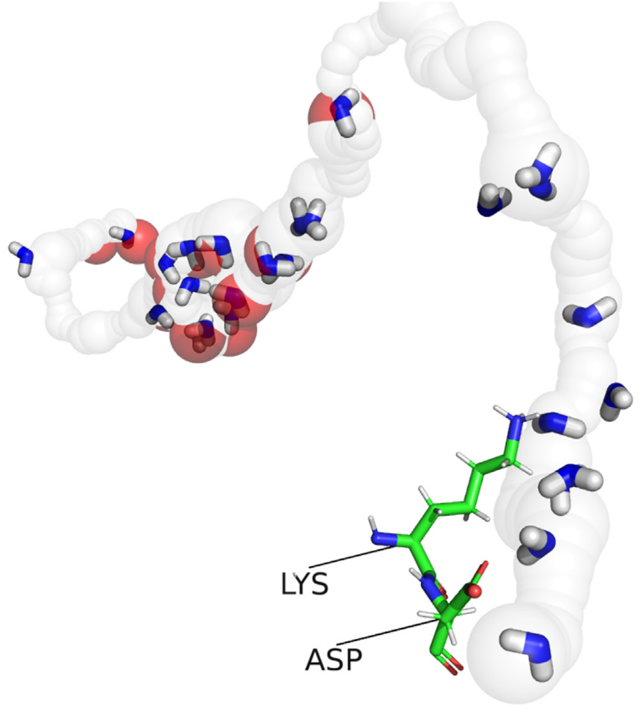

FIGURE 10 At the exit of central channel in subunit L, there are two crucial residues, LYS396 and ASP397. Mutations of these two residues are known to significantly reduce proton translocation (7). We have found water molecules in this region using Dowser++, shown as blue and white sticks. Experimental water molecules are shown as red spheres.

Table 1 and Fig. 13 present the key results of the analysis. Here, we will assume the open structure of *Y. lipolytica* 7o71 (20), where the E-channel is disconnected from the central axis. However, as we pointed out in effect of mutation in the gap region of E-channel (TM3/J/ND6), in the assumed closed structure, the isolating gap is possibly present too.

The connectivity between charged residues along the network of water channels

The connectivity was defined simply by the cutoff distance for proton transfer along the path, which was 3.5 Å between the oxygen atoms of the water molecules or those of water and possible intermediate protonatable protein residues. This was possible to do in a direct and detailed manual analysis.

The membrane structure is connected horizontally and vertically through protonatable residues and water molecules as detailed in Table 1 and summarized in Fig. 13.

There are "patches" of connectivity inside the membrane. Fig. 13 shows that gaps exist between the patches of connected segments. A gap is defined as an area where no water was found experimentally and theoretically, the space is too small to accommodate water molecules, and no protein residues can be continuously connected without

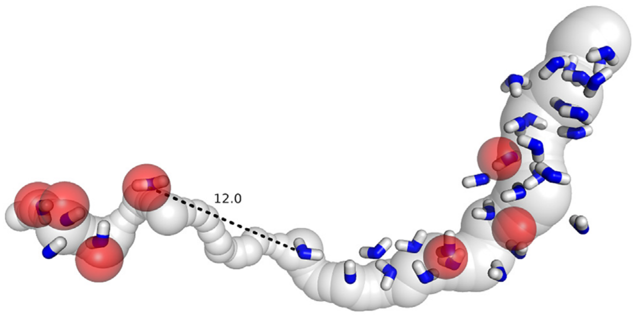

FIGURE 11 7 experimental (*red spheres*) and ∼30 Dowser++-calculated (*blue sticks*) water molecules in the E-channel of charged subunits J, K, N, A, and H. There clearly exists a gap in water connectivity in the explored structure 7o71 (20), shown here in the central part of the structure, that corresponds to the (TM3/ND6/J) region of the enzyme structure. Presumably, this is characteristic of only an "open" state, while in the "closed" state, the gap is hydrated (17,22).

water. There are at least three such gaps in the central part of the channel (i.e., in *Y. lipolytica* PDB: 7O71 (20) structure).

This network provides an insight on how the protons may move around in the membrane domain. The key conclusion is that protons can move horizontally, through water chains and charged residues along the central axis, starting from the quinone binding cavity. However, the gaps between patches of the channel would stop protons from moving between the patches. Although protons cannot be transferred across the gaps, there is a Coulomb interaction between the protons of neighboring patches; this interaction can play a crucial role for the pumping mechanism of the enzyme (28,29). One possible mechanism of pumping that combines proton-conducting patches and isolating Coulomb junctions was recently proposed by our group (29) and was also discussed elsewhere (18,28). Here, we will limit the mechanistic discussion by these brief statements, keeping the paper focused on the hydration subject. Obviously, the proton transfer details, as well as the mechanism of pumping itself, is a complicated subject that needs to be addressed in a separate, more focused study, which will be described elsewhere.

CONCLUSIONS

We have explored a strategy to identify potential proton transfer channels in a protein structure and applied it to *Y. lipolytica* respiratory complex I. The procedure is based on the Caver program for Voronoi partitioning of the protein structure and identification of connected voids in the structure that form channels, with subsequent hydration of the

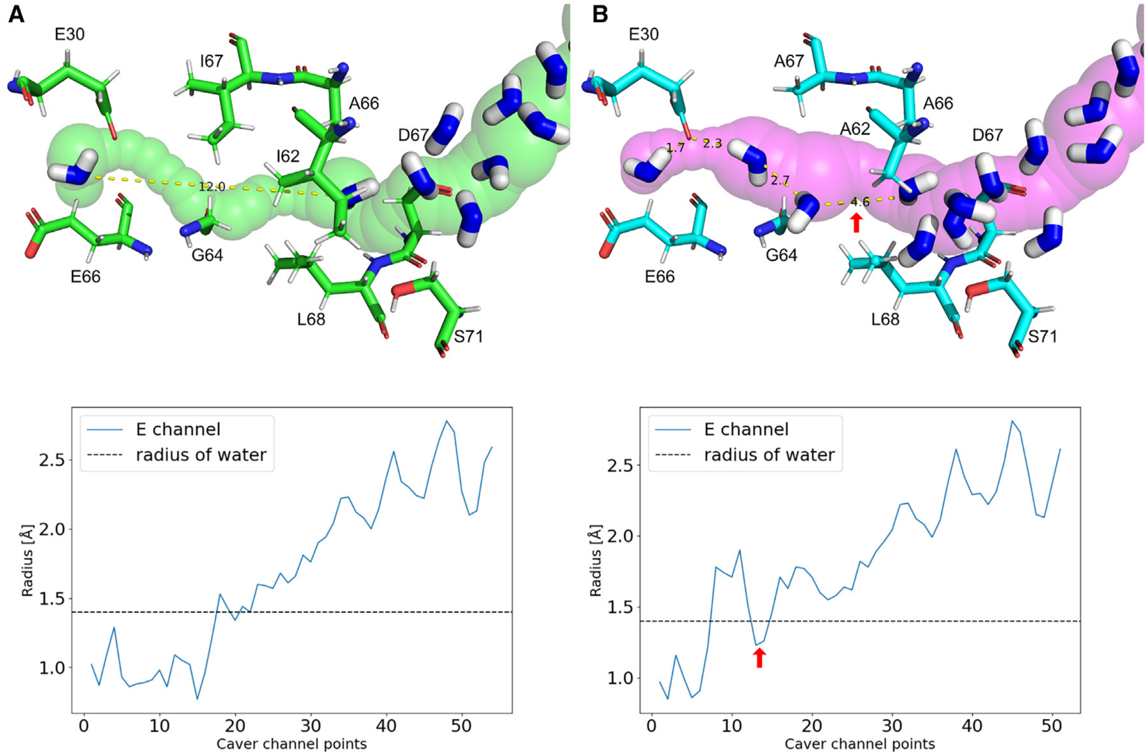

FIGURE 12 Result of mutation of ILE 62 and ILE 67 into ALA 62 and ALA 67 in the gap region. Shown on the left (*A*) is the channel gap between subunits A and K calculated using the structure of *Y. lipolytica* 7o71 (20). The channel on the right (*B*) is calculated after mutating ILE 62 and ILE 67 into alanines. Shown here is the charged structure, and results for the neutral structure are shown in the supporting material. The arrow in (*B*) indicates the remaining hydrophobic gap (4.6 Å for charged, and 6.0 Å for neutral structure).

TABLE 1 Connectivity of the central axis residues

Subunit	Residue	Connectivity to left	Connectivity to right	Connectivity to top	Connectivity to bottom
L	LYS511	none	ASP397	none	surface
L	ASP397	LYS511	LYS396	none	surface
L	LYS396	ASP397	none	LYS339	none
L	LYS339	LYS396 (via SER308-TYR428-SER311-W1W2-GLN312)	HIS251	none	none
L	LYS302	none	HIS251	surface	none
L	HIS251	LYS302, LYS339 (via THR309)	none	narrow channel	none
L	LYS226	none (G12.0)	ASP178	narrow channel	none
L	ASP178	LYS226	GLU144	narrow channel	narrow channel
L	GLU144	ASP178	ARG175	narrow channel	narrow channel
L	ARG175	GLU144	GLU395-M	narrow channel	narrow channel
M	GLU395	ARG175-L	HIS309	water channel	narrow channel
M	HIS309	GLU395	HIS335	water channel	narrow channel
M	HIS335	HIS309	LYS252	water channel	narrow channel
M	LYS252	HIS335	none (G10.7)	none	dry channel
M	LYS221	none (G10.7)	GLU142	none	none
M	GLU142	LYS221	LYS383-N (G4.2)	none	none
N	LYS383	GLU142-M	HIS320	dry channel	dry channel
N	HIS320	LYS383 (G7.5)	LYS241	water channel	none
N	LYS241	HIS320	LYS211	water channel	none
N	LYS211	LYS241	GLU131	none	none
N	GLU131	LYS211	GLU66-K	none	dry channel
K	GLU66	GLU131-N	GLU30	water channel	narrow channel
K	GLU30	GLU66	none (G13.8)	water channel	narrow channel
A	ASP67	none (G13.8)	GLU147-H	none	SER71
H	GLU147	ASP67	GLU196	none	none
H	GLU196	GLU147	GLU231	none	none
H	GLU231	GLU196	none	none	none

"Left" and "right" correspond to a position along the central channel with respect to a give residue. "Top" is the N-side, and "bottom" is the P-side of the membrane. A narrow channel is a channel that has water molecules with bottlenecks or gaps. A dry channel is a channel that is just short of enough space for water molecules where no water molecules were found. A water channel is a channel that has water connectivity throughout the channel. The letter "G" stands for a "gap," followed by the length of the gap in angstroms. A gap is a region where no water molecules were found and the space is too small to accommodate water molecules.

found channels by the water placement program D++. Most channels are very narrow and can accommodate only single-file water chains or none. The nonuniform distribution of bound water reveals that proton conductivity in these channels is highly variable.

The accurate characterization of conductivity requires further analysis of each of the potential proton transfer channels; however, it is already clear, qualitatively, that some channels are open for proton transfer while others are closed but could be opened with relatively minor changes in the protein structure.

It is important that we have identified an apparently complete set of potential channels, which, one by one, can be characterized by proton conductivity and their possible modulation/gating by the structural changes.

For a given structure 7o71 (20) of the enzyme, it is clear there are gaps in the central axis channel. As shown in Table 1 and Fig. 13, three or possibly four gaps are well resolved. This remains a true event in the more hydrated closed structure (see discussion in effect of mutation in the gap region of E-channel (TM3/J/ND6)). But more gaps can be expected, as proton conductivity can depend on minor details; it is a challenging issue that will be addressed elsewhere. The present study suggests that the central axis channel consists of proton-conducting patches, within which protons can move, separated by the isolating gaps. This picture is in agreement with the assumption of separate (up to four) pumping units in the enzyme (29), and it is in conflict with the assumption that only the L/ND5 subunit expels all four pumped protons by a continuous central axis channel that exits on the P-side (28) (see Fig. 1).

The upper part of the membrane surface (N-side) in Fig. 1 is seen to be much more connected to the central channel than the lower part (P-side). This is in agreement with previous analyses of the structure of complex I (23,28); however, here, we have identified a number of half-channels leading to the P-side of the membrane, which can be classified (in a given structure) as closed. In our classification, these are narrow channels, which are partially hydrated but have significant isolating gaps; there are also un-hydrated, dry channels. It is clear that these channels are in the closed states for a given structure but possibly can be opened by relatively small changes in the structure; the changes can be transient in nature and naturally difficult to observe. In any case, keeping in mind that proton transfer channels require gating, i.e., modulation to function as

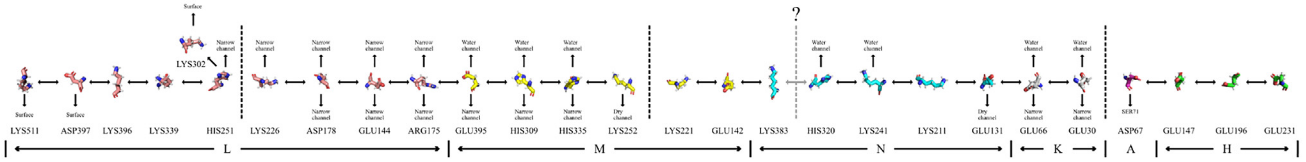

FIGURE 13 Connectivity of the central axis residues in *Y. lipolytica* complex I, PDB: 7o71. The gaps of connectivity are shown as vertical dashed lines. The gray dashed line with a question mark is an uncertain gap that can be possibly filled with water or connected otherwise.

pumping gears, it is quite clear that a single snapshot of the structure (PDB: 7o71 (20) studied here) is highly unlikely to reveal all stages of the transient system in action. Overall, most of the qualitative conclusions based on the predictions here are in line with those derived from the analysis of empirical cryo-EM data for water in mouse complex I reported recently (17), demonstrating the usefulness of the approach and providing an alternative avenue of exploring proton transfer channels compared to the usual hydrogen bond network analysis (40).

Obviously, the present study does not provide a complete or final picture of the pump; however, one should not overlook that we now have a complete set of potential proton channels, which can be characterized, one by one, in a more focused and detailed study of their conductivity. This is important, as proton transfer pathways are usually not well defined. A detailed characterization of proton conductivity in the identified channels is the next step in our program, which will be reported elsewhere.

DATA AND CODE AVAILABILITY

All data generated or analyzed during this study are included in this published article and its supporting material file.

SUPPORTING MATERIAL

Supporting material can be found online at https://doi.org/10.1016/j.bpj.2024.07.041.

AUTHOR CONTRIBUTIONS

P.W., J.D., and S.M. developed methodology of using Caver for searching putative proton transfer channels and applied it in this project; P.W. ran D++ and MD simulations of water placement in the channels and studied the connectivity of the channels; A.A.S. designed the research; A.A.S. and P.W. wrote the paper.

ACKNOWLEDGMENTS

This work has been supported by the NIH research grant GM054052 (A.A.S.).

DECLARATION OF INTERESTS

The authors declare no competing interests.

REFERENCES

1. Sazanov, L. A. 2015. A giant molecular proton pump: structure and mechanism of respiratory complex I. *Nat. Rev. Mol. Cell Biol.* 16:375–388.
2. Hirst, J., and M. M. Roessler. 2016. Energy conversion, redox catalysis and generation of reactive oxygen species by respiratory complex I. *Biochim. Biophys. Acta.* 1857:872–883.
3. Parey, K., C. Wirth, ..., V. Zickermann. 2020. Respiratory complex I - structure, mechanism and evolution. *Curr. Opin. Struct. Biol.* 63:1–9.
4. Hayashi, T., and A. A. Stuchebrukhov. 2010. Electron tunneling in respiratory complex I. *Proc. Natl. Acad. Sci. USA.* 107:19157–19162.
5. Hirst, J. 2005. Energy transduction by respiratory complex I-an evaluation of current knowledge. *Biochem. Soc. Trans.* 33:525–529.
6. Wikström, M. 2017. Mechanisms of Primary Energy Transduction in Biology. Royal Society of Chemistry.
7. Fiedorczuk, K., and L. A. Sazanov. 2018. Mammalian Mitochondrial Complex I Structure and Disease-Causing Mutations. *Trends Cell Biol.* 28:835–867.
8. Baradaran, R., J. M. Berrisford, ..., L. A. Sazanov. 2013. Crystal structure of the entire respiratory complex I. *Nature.* 494:443–448.
9. Fiedorczuk, K., J. A. Letts, ..., L. A. Sazanov. 2016. Atomic structure of the entire mammalian mitochondrial complex I. *Nature.* 538:406–410.
10. Gutierrez-Fernandez, J., K. Kaszuba, ..., L. A. Sazanov. 2020. Key role of quinone in the mechanism of respiratory complex I. *Nat. Commun.* 11:4135.
11. Zickermann, V., C. Wirth, ..., U. Brandt. 2015. Structural biology. Mechanistic insight from the crystal structure of mitochondrial complex I. *Science.* 347:44–49.
12. Parey, K., O. Haapanen, ..., V. Zickermann. 2019. High-resolution cryo-EM structures of respiratory complex I: Mechanism, assembly, and disease. *Sci. Adv.* 5:eaax9484.
13. Agip, A. N. A., J. N. Blaza, ..., J. Hirst. 2019. Mammalian Respiratory Complex I Through the Lens of Cryo-EM. *Annu. Rev. Biophys.* 48:165–184.
14. Guo, R., S. Zong, ..., M. Yang. 2017. Architecture of Human Mitochondrial Respiratory Megacomplex. *Cell.* 170:1247–1257.e12.
15. Bridges, H. R., J. G. Fedor, ..., J. Hirst. 2020. Structure of inhibitor-bound mammalian complex I. *Nat. Commun.* 11:5261.
16. Grba, D. N., and J. Hirst. 2020. Mitochondrial complex I structure reveals ordered water molecules for catalysis and proton translocation. *Nat. Struct. Mol. Biol.* 27:892–900.
17. Grba, D. N., I. Chung, ..., J. Hirst. 2023. Investigation of hydrated channels and proton pathways in a high-resolution cryo-EM structure of mammalian complex I. *Sci. Adv.* 9:eadi1359.
18. Kampjut, D., and L. A. Sazanov. 2020. The coupling mechanism of mammalian respiratory complex I. *Science.* 370:eabc4209.
19. Gu, J., T. Liu, ..., M. Yang. 2022. The coupling mechanism of mammalian mitochondrial complex I. *Nat. Struct. Mol. Biol.* 29:172–182.
20. Parey, K., J. Lasham, ..., V. Zickermann. 2021. High-resolution structure and dynamics of mitochondrial complex I-Insights into the proton pumping mechanism. *Sci. Adv.* 7:eabj3221.

21. Kravchuk, V., O. Petrova, …, L. Sazanov. 2022. A universal coupling mechanism of respiratory complex I. *Nature*. 609:808–814.
22. Laube, E., J. Meier-Credo, …, W. Kühlbrandt. 2022. Conformational changes in mitochondrial complex I of the thermophilic eukaryote Chaetomium thermophilum. *Sci. Adv.* 8:eadc9952.
23. Kampjut, D., and L. A. Sazanov. 2022. Structure of respiratory complex I - An emerging blueprint for the mechanism. *Curr. Opin. Struct. Biol.* 74:102350.
24. Vercellino, I., and L. A. Sazanov. 2022. The assembly, regulation and function of the mitochondrial respiratory chain. *Nat. Rev. Mol. Cell Biol.* 23:141–161.
25. Kaila, V. R. I. 2021. Resolving Chemical Dynamics in Biological Energy Conversion: Long-Range Proton-Coupled Electron Transfer in Respiratory Complex I. *Acc. Chem. Res.* 54:4462–4473.
26. Kaila, V. R. I. 2018. Long-range proton-coupled electron transfer in biological energy conversion: towards mechanistic understanding of respiratory complex I. *J. R. Soc. Interface.* 15:20170916.
27. Kaila, V. R. I., M. Wikström, and G. Hummer. 2014. Electrostatics, hydration, and proton transfer dynamics in the membrane domain of respiratory complex I. *Proc. Natl. Acad. Sci. USA.* 111:6988–6993.
28. Sazanov, L. A. 2023. From the 'black box' to 'domino effect' mechanism: what have we learned from the structures of respiratory complex I. *Biochem. J.* 480:319–333.
29. Stuchebrukhov, A. A., and T. Hayashi. 2023. Single protonation of the reduced quinone in respiratory complex I drives four-proton pumping. *FEBS Lett.* 597:237–245.
30. Djurabekova, A., J. Lasham, …, V. Sharma. 2024. Long-range electron proton coupling in respiratory complex I — insights from molecular simulations of the quinone chamber and antiporter-like subunits. *Biochem. J.* 481:499–514.
31. Djurabekova, A., O. Haapanen, and V. Sharma. 2020. Proton motive function of the terminal antiporter-like subunit in respiratory complex I. *Biochim. Biophys. Acta Bioenerg.* 1861:148185.
32. Wang, P., N. Dhananjayan, …, A. A. Stuchebrukhov. 2021. Respiratory complex I: Bottleneck at the entrance of quinone site requires conformational change for its opening. *Biochim. Biophys. Acta Bioenerg.* 1862:148326.
33. Dhananjayan, N., P. Wang, …, A. A. Stuchebrukhov. 2022. Quinone binding in respiratory complex I: Going through the eye of a needle. The squeeze-in mechanism of passing the narrow entrance of the quinone site. *Photochem. Photobiol. Sci.* 21:1–12.
34. Wikstrom, M., A. Djurabekova, and V. Sharma. 2023. On the role of ubiquinone in the proton translocation mechanism of respiratory complex I. *FEBS Lett.* 597:224–236.
35. Hoias Teixeira, M., and G. Menegon Arantes. 2019. Balanced internal hydration discriminates substrate binding to respiratory complex I. *Biochim. Biophys. Acta Bioenerg.* 1860:541–548.
36. Verkhovskaya, M., and D. A. Bloch. 2013. Energy-converting respiratory Complex I: on the way to the molecular mechanism of the proton pump. *Int. J. Biochem. Cell Biol.* 45:491–511.
37. Di Luca, A., A. P. Gamiz-Hernandez, and V. R. I. Kaila. 2017. Symmetry-related proton transfer pathways in respiratory complex I. *Proc. Natl. Acad. Sci. USA.* 114:E6314–E6321.
38. Muhlbauer, M. E., P. Saura, …, V. R. I. Kaila. 2020. Water-Gated Proton Transfer Dynamics in Respiratory Complex I. *J. Am. Chem. Soc.* 142:13718–13728.
39. Chovancova, E., A. Pavelka, …, J. Damborsky. 2012. CAVER 3.0: a tool for the analysis of transport pathways in dynamic protein structures. *PLoS Comput. Biol.* 8:e1002708.
40. Khaniya, U., C. Gupta, …, M. R. Gunner. 2020. Hydrogen bond network analysis reveals the pathway for the proton transfer in the E-channel of T. thermophilus Complex I. *Biochim. Biophys. Acta Bioenerg.* 1861:148240.
41. Bertalan, É., S. Lešnik, …, A. N. Bondar. 2020. Protein-water hydrogen-bond networks of G protein-coupled receptors: Graph-based analyses of static structures and molecular dynamics. *J. Struct. Biol.* 212:107634.
42. Stuchebrukhov, A. A. 2009. Mechanisms of proton transfer in proteins: Localized charge transfer versus delocalized soliton transfer. *Phys. Rev. E.* 79:031927.
43. Piana, S., A. G. Donchev, …, D. E. Shaw. 2015. Water Dispersion Interactions Strongly Influence Simulated Structural Properties of Disordered Protein States. *J. Phys. Chem. B.* 119:5113–5123.
44. Morozenko, A., and A. A. Stuchebrukhov. 2016. Dowser++, a new method of hydrating protein structures. *Proteins.* 84:1347–1357.
45. Farahvash, A., and A. Stuchebrukhov. 2018. Investigating the Many Roles of Internal Water in Cytochrome c Oxidase. *J. Phys. Chem. B.* 122:7625–7635.
46. Efremov, R. G., and L. A. Sazanov. 2011. Respiratory complex I: 'steam engine' of the cell? *Curr. Opin. Struct. Biol.* 21:532–540.
47. Yi, J. T., P. Wang, and A. A. Stuchebrukhov. 2023. Mutation at the entrance of the quinone cavity severely disrupts quinone binding in respiratory complex I. *Sci. Rep.* 13:20413.
48. Chung, I., D. N. Grba, …, J. Hirst. 2022. Making the leap from structure to mechanism: are the open states of mammalian complex I identified by cryoEM resting states or catalytic intermediates? *Curr. Opin. Struct. Biol.* 77:102447.
49. Grba, D. N., J. J. Wright, …, J. Hirst. 2024. Molecular mechanism of the ischemia-induced regulatory switch in mammalian complex I. *Science.* 384:1247–1253.
50. Morozenko, A., I. V. Leontyev, and A. A. Stuchebrukhov. 2014. Dipole Moment and Binding Energy of Water in Proteins from Crystallographic Analysis. *J. Chem. Theor. Comput.* 10:4618–4623.
51. Abraham, M. J., T. Murtola, …, E. Lindahl. 2015. GROMACS: High performance molecular simulations through multi-level parallelism from laptops to supercomputers. *SoftwareX.* 1–2:19–25.
52. Leontyev, I., and A. Stuchebrukhov. 2011. Accounting for electronic polarization in non-polarizable force fields. *Phys. Chem. Chem. Phys.* 13:2613–2626.
53. Lasham, J., A. Djurabekova, …, V. Sharma. 2024. Role of Protonation States in the Stability of Molecular Dynamics Simulations of High-Resolution Membrane Protein Structures. *J. Phys. Chem. B.* 128:2304–2316.
54. Quenneville, J., D. M. Popović, and A. A. Stuchebrukhov. 2006. Combined DFT and electrostatics study of the proton pumping mechanism in cytochrome c oxidase. *Biochim. Biophys. Acta.* 1757:1035–1046.
55. Couch, V., D. Popovic, and A. Stuchebrukhov. 2011. Redox-coupled protonation of respiratory complex I: the hydrophilic domain. *Biophys. J.* 101:431–438.
56. Stuchebrukhov, A. A., and D. M. Popovic. 2006. Comment on "Acidity of Cu-bound histidine in the binuclear center of cytochrome oxidase. *J. Phys. Chem. B.* 110:17286–17287.
57. Trott, O., and A. J. Olson. 2010. AutoDock Vina: improving the speed and accuracy of docking with a new scoring function, efficient optimization, and multithreading. *J. Comput. Chem.* 31:455–461.
58. Zdorevskyi, O., A. Djurabekova, …, V. Sharma. 2023. Horizontal proton transfer across the antiporter-like subunits in mitochondrial respiratory complex I. *Chem. Sci.* 14:6309–6318.

Biophysical Journal, Volume *123*

Supplemental information

Searching for proton transfer channels in respiratory complex I

Panyue Wang, Jackson Demaray, Stanislav Moroz, and Alexei A. Stuchebrukhov

Supplementary Information

Searching for Proton Transfer Channels in Respiratory Complex I

Panyue Wang, Jackson Demaray‡, Stanislav Moroz‡, and Alexei A. Stuchebrukhov*

Department of Chemistry, University of California at Davis, One Shields Avenue, Davis, California 95616

*Corresponding author: Alexei A. Stuchebrukhov,
Department of Chemistry, University of California at Davis.
E-mail: aastuchebrukhov@ucdavis.edu

‡These authors contributed equally.

0. Overall structure of the central axis water channel

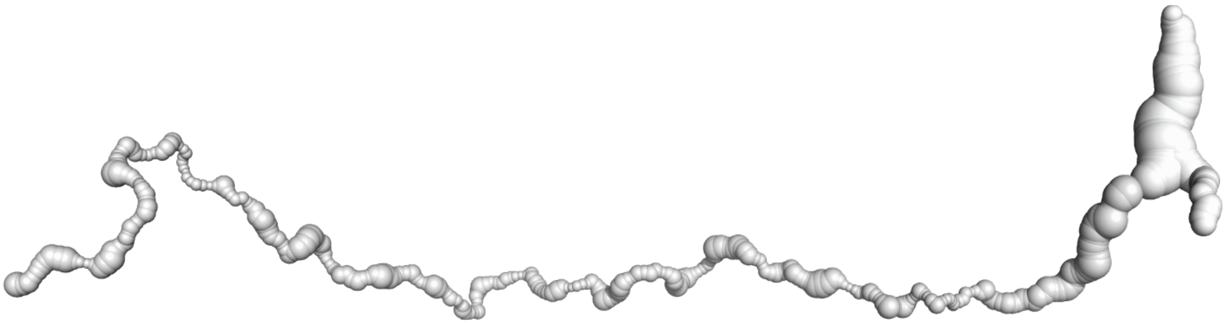

Figure S0. The proton transfer channel along the central axis with the quinone binding cavity shown on the right side of the figure. For better orientation, see Fig. 1 and 2 of the main text.

1. Central membrane part, subunits M and N

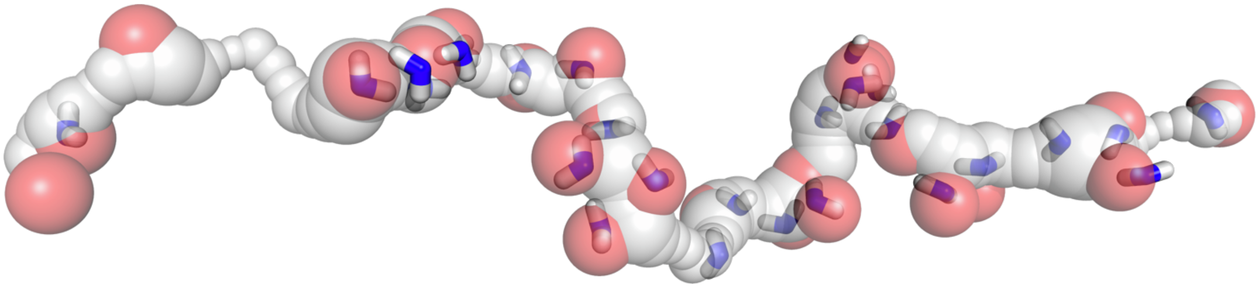

Figure S1. 26 experimental (red spheres) and 23 Dowser++ calculated (blue sticks) water molecules in the channel of *neutral* subunits M and N.

2. Left-most part (exit) of central axis, subunits L and M

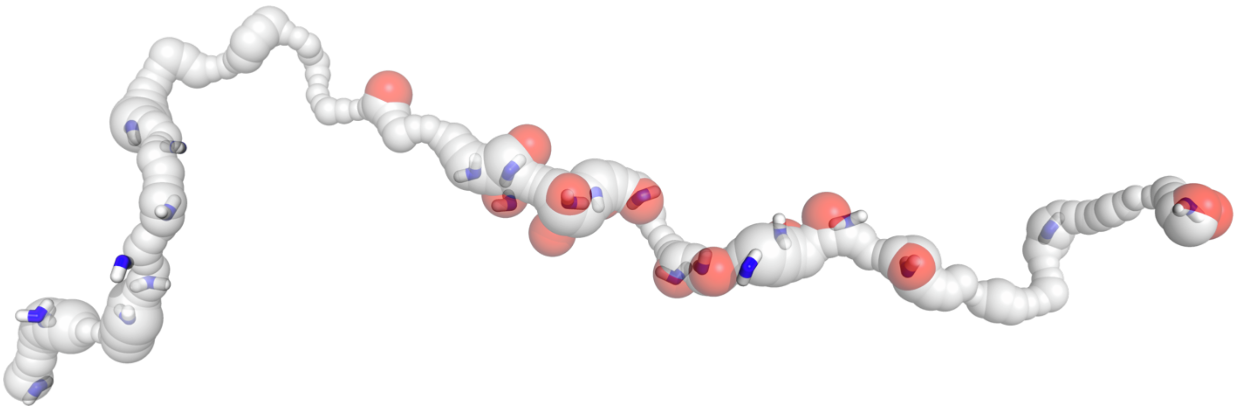

Figure S2. 14 experimental (red spheres) and 23 Dowser++ calculated (blue sticks) water molecules in the channel of *neutral* subunit L and M. For better orientation, see Fig. 1 and 2 of the main text.

3. The entrance of central axis: E-channel and JK interface. Detailed study of hydration

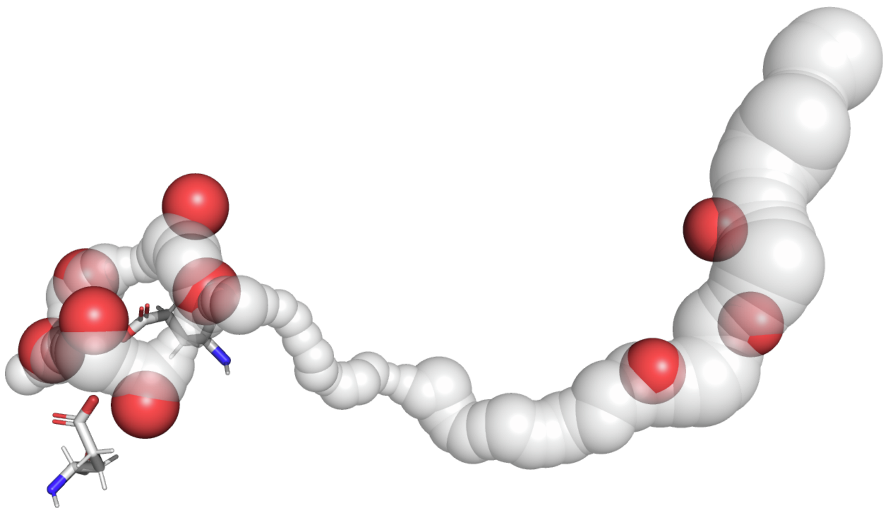

Figure S3. A noticeable difference in the structure of the E channel in two charged sates at JK interface (left-most part). Here shown is the channel structure that incorporates *both* charged and neutral states. 9 experiment water molecules are seen in the *combined* E channel. Two glutamic acids charged states result in a loop structure of the channel with experimentally resolved water molecules. This is an example of PT brunching structure of pathways/channels.

4. Comparing Dowser++ and MD energies of water insertion/removal

4.1. Using TIP3PP model of Dowser++, standard protonation states of the protein.

Shown in Figs. S4a and S4b below are three different ways to estimate binding energy of Dowser++ (D++) inserted water molecules.

A) Red - the original Dowser++ (D++) energy.

In D++, TIP3PP charges are scaled by factor 0.8 compared with TIP3P to reflect the less polar character of water in protein, TIP3PP, compared with the bulk model, TIP3P. After "draining", no interaction between water molecules are included, only interaction with the protein is counted. No relaxation of the inserted water molecules, nor the protein structure, is involved, except for rotational equilibration of hydrogen bonds [1-3].

B) Blue – Gromacs re-evaluation of the average potential energy of the inserted water molecules with TIP3PP model. This is the same water model as in D++ (TIP3P for Protein, TIP3PP model; charges are scaled by a factor 0.8). Both relaxation of water clusters and protein matrix are included. The average potential energy is evaluated along a 3ns dynamic trajectory of local structure.

C) Green - The binding energy is evaluated with Gromacs as in B), but now as a difference E(N)-E(N-1)_i of energies of two clusters with N and (N-1) water molecules upon removal of *i-th* water molecule from the cluster. The original cluster of N-molecules is formed by D++ procedure. The calculated energy value (with negative sign) corresponds to binding energy (work to remove a given water from the site), except that entropy is not counted. This energy is to be compared with the corresponding value in the bulk water -10kcal/mol. The remaining entropy change and the change in kinetic energy can amount to as much as 5kcal/mol. Thus, the actual cutoff energy is somewhere between -10 and 5 kcal/mol. The empirical threshold based on the analysis of experimental data of available structures is shown to be about -5kcal/mol.[1-3]

1. Morozenko, A.; Stuchebrukhov, A. A., Dowser++, a new method of hydrating protein structures. *Proteins* **2016,** *84* (10), 1347-57.

2. Morozenko, A.; Leontyev, I. V.; Stuchebrukhov, A. A., Dipole Moment and Binding Energy of Water in Proteins from Crystallographic Analysis. *J. Chem. Theory Comput.* **2014,** *10* (10), 4618-4623.

3. Farahvash, A.; Stuchebrukhov, A., Investigating the Many Roles of Internal Water in Cytochrome c Oxidase. *J. Phys. Chem. B* **2018,** *122* (31), 7625-7635.

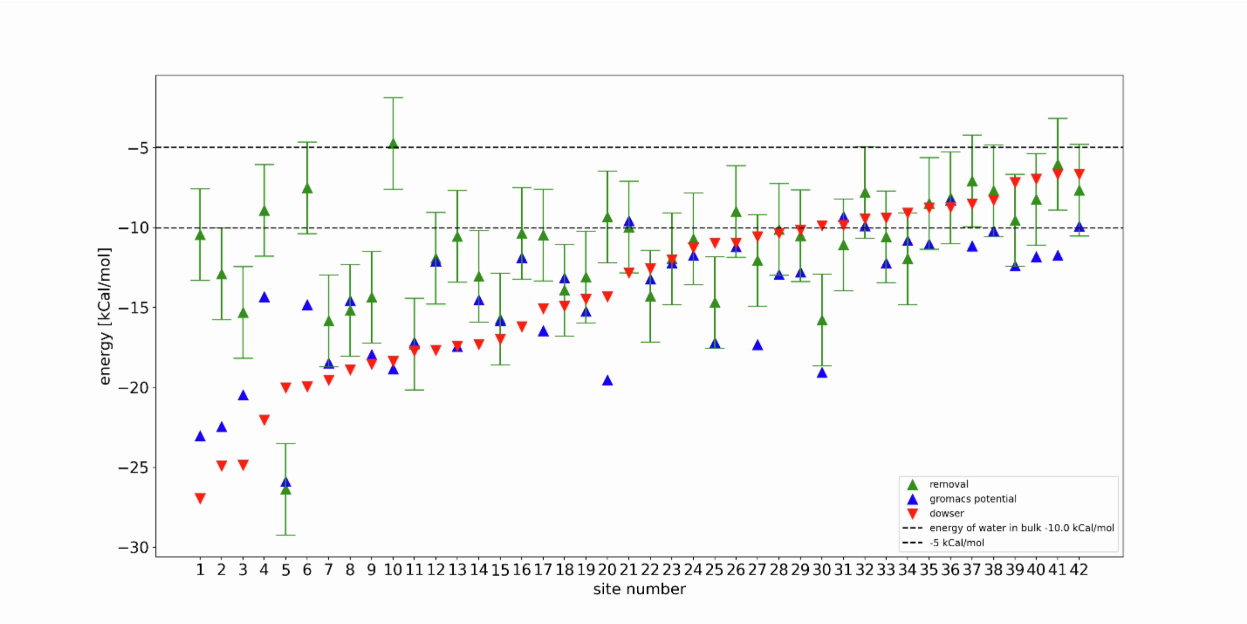

Figure S4a. Binding (removal) energy of Dowser++ water molecules in *charged* structure of extended E channel including JK subunits (see Figs. below). All Dowser++ inserted molecules were re-evaluated with Gromacs and TIP3PP model and remain below the threshold of -5kcal/mol.

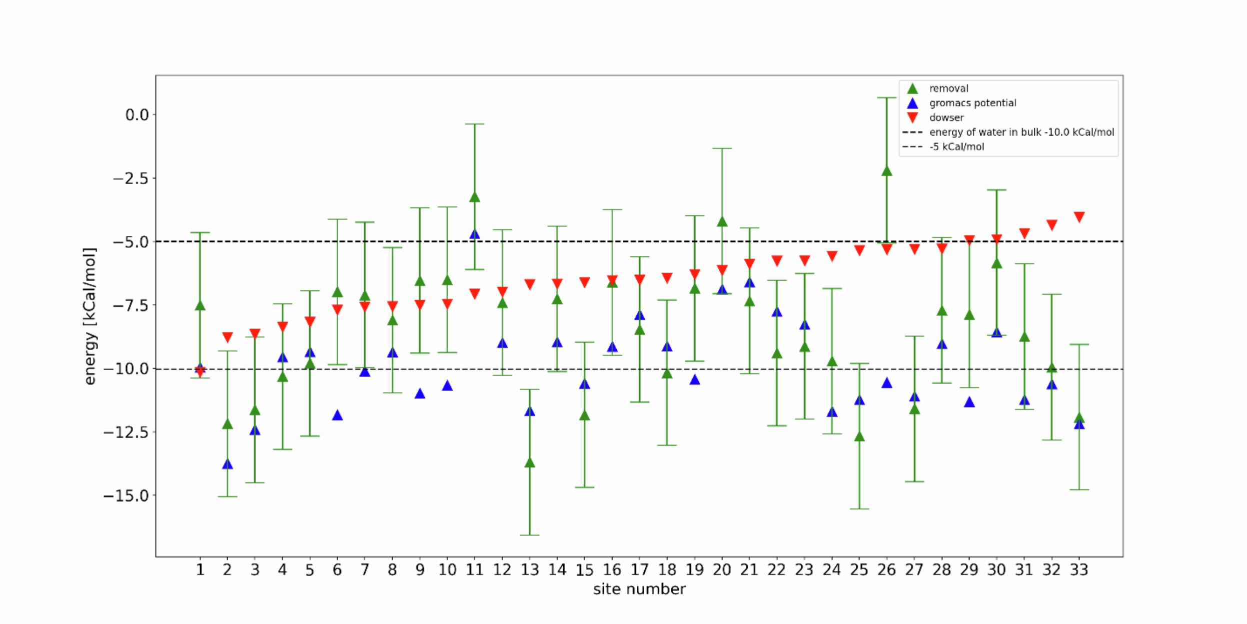

Figure S4b. Binding (removal) energy of Dowser++ water molecules in the *neutral* structure. Same as in Fig. 4a, but for neutral structure. Molecule i=11 is high in energy due to relaxation of the structure, 20 and 26 are due to HB clashes, removal of one stabilizes the other.

4.2. Effect of protein charge scaling.

Here we explored the effect of charge-scaling of protein charged residues, see Ref. [45] of the main text, Leontyev, I.; Stuchebrukhov, A., Accounting for electronic polarization in non-polarizable force fields. *Phys Chem Chem Phys* **2011,** *13* (7), 2613-2626. **Figs. S5-7** are informative as they give the scale of changes that occur upon the reduction (by a factor of ~0.7) of protein charges.

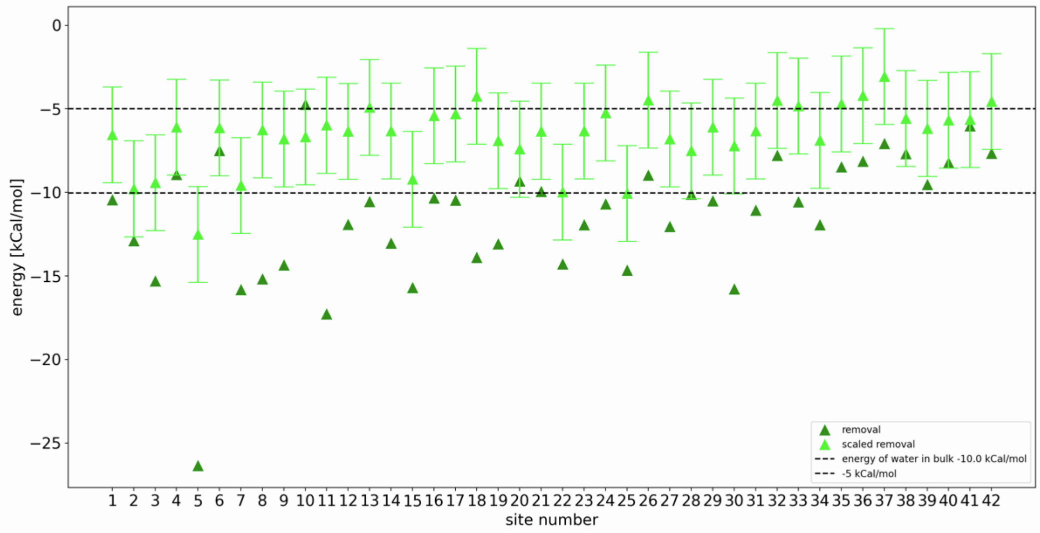

Figure S5. Binding (removal) energy of Dowser water molecules in *charged* structure of extended E channel including JK subunits. The charges of protonatable side chains are scaled (0.7) in calculations shown in light green. Almost all binding energies have increased except for water *i*=10 where scaled binding energy is ~2kcal/mol lower than unscaled calculations. This difference is within fluctuations shown as error bars in this graph.

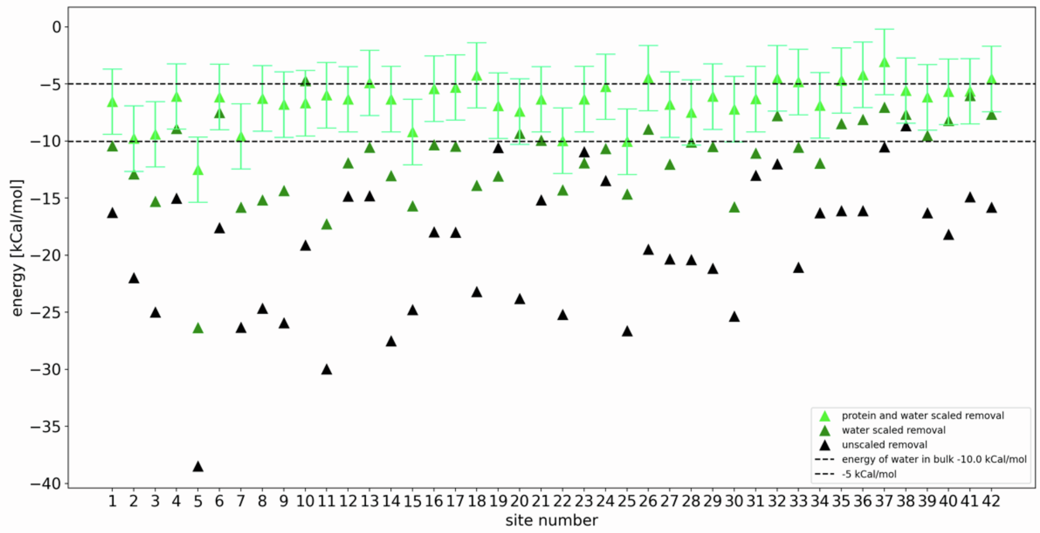

Figure S6. Binding (removal) energy of Dowser water molecules in *charged* structure of extended E channel including JK subunits. Both charges of protonatable side chains and charges of water are scaled in calculations shown in light green. The charges of water are scaled (TIP3PP) in calculations shown in dark green, while the charges of the protonatable side chains remain unscaled. For calculations shown in black, the charges of protonatable side chains and water are unscaled, which is the default setting of the CHARMM36 forcefield.

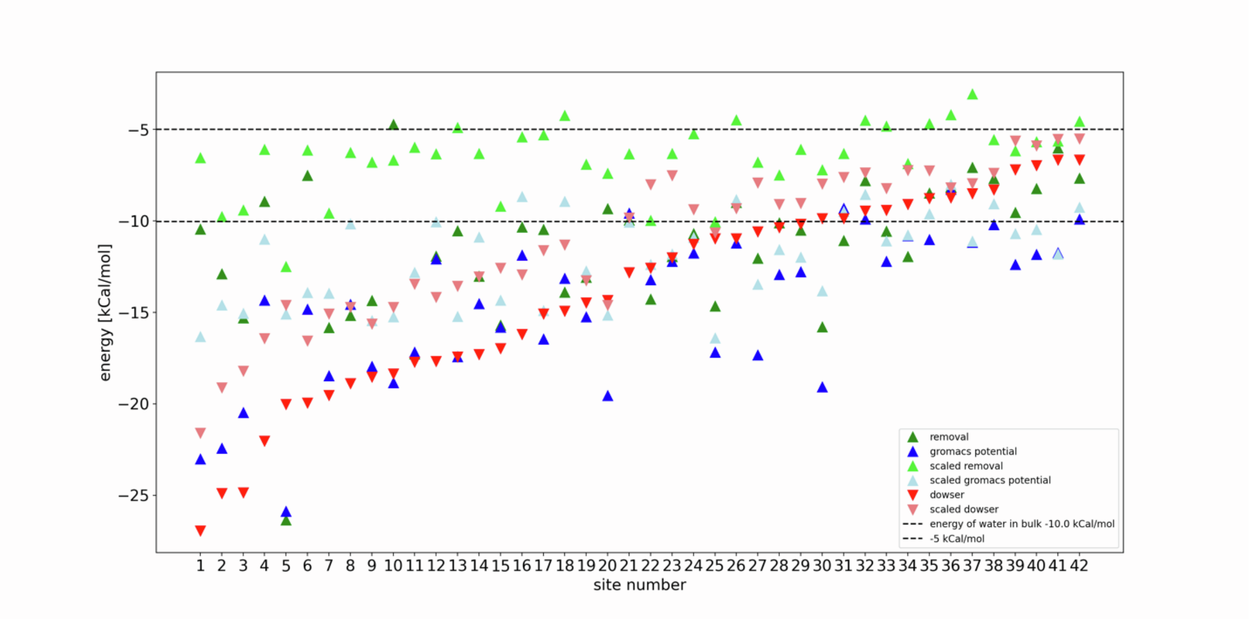

Figure S7. Summary of binding energy of water molecules in *charged* structure of extended E channel (including JK subunits) calculated by all 6 different schemes. The charges of protonatable side chains are scaled in calculations shown in light green, light blue and coral. The absolute energies of all schemes are rather different, but all still mostly below the likely threshold of -5kcal/mol.

5. E-channel hydration. Different hydration models predictions

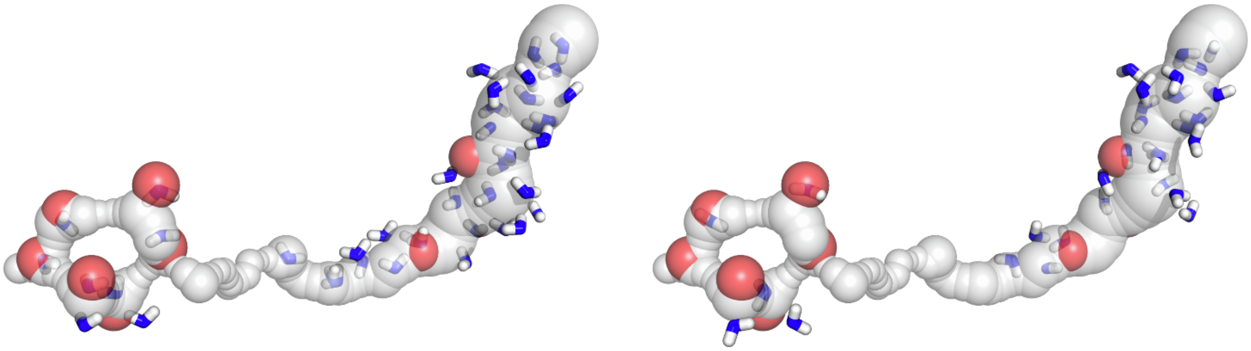

Figure S8a. 9 experimental (red spheres) and 41 Dowser++ calculated (blue sticks) water molecules in the charged (left) extended E-channel of subunits J, K, A and H vs. 30 Dowser++ calculated water molecules in the neutral (right) E-channel with removal energies lower than -5 kcal/mol. Notice, predicted water in the charged state of JK part of the channel (leftmost part) better matches experimental position of water molecules. Whereas predictions in the proper E-

channel (rightmost part, after the gap in water chain) apparently better match experiment in the neutral state, as the charged state grossly exceeds the observed 3 experimental water molecules for the whole range of possible cutoffs (5-10 kcal/mol) shown in Figs. S4-S7.

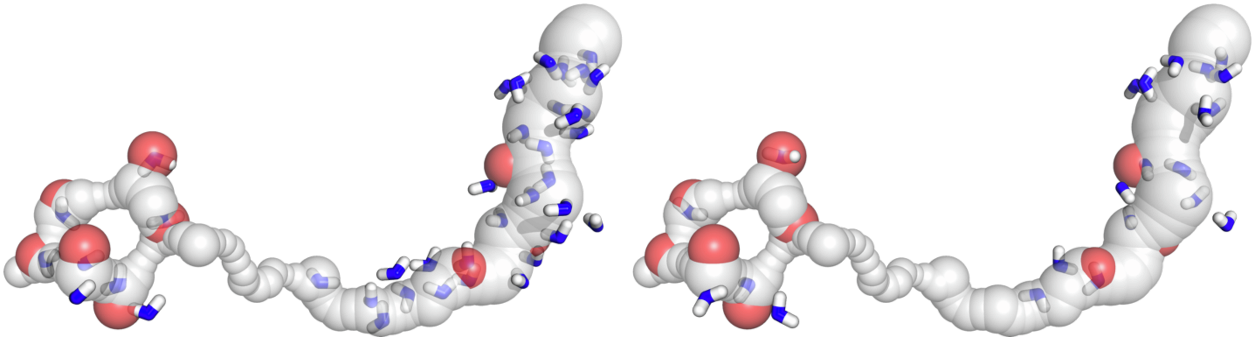

Figure S8b. 9 experimental (red spheres) and 39 Dowser++ calculated (blue sticks) water molecules in the charged (left) E-channel of subunits J, K, N, A and H vs. 20 Dowser++ calculated water molecules in the neutral (right) E-channel with removal energies lower than -7.5 kcal/mol.

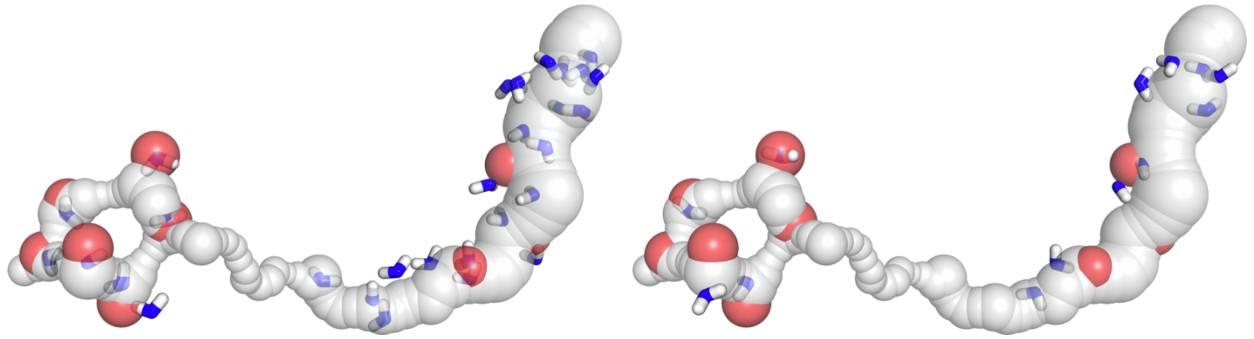

Figure S8c. 9 experimental (red spheres) and 27 Dowser++ calculated (blue sticks) water molecules in the charged (left) E-channel of subunits J, K, N, A and H vs. 9 Dowser++ calculated water molecules in the neutral (right) E-channel with removal energies lower than -10 kcal/mol.

Overall, our simulations indicate that the actual number of hydrated water molecules in the E-channel significantly exceeds those few that were observed in the cryo-EM structure of *Y. lipolytica* 7o71[20] but in line with, e.g., data for mouse complex I (see Ref. [17] in the main text, Grba, D. N.; Chung, I.; Bridges, H. R.; Agip, A. A.; Hirst, J., Investigation of hydrated channels and proton pathways in a high-resolution cryo-EM structure of mammalian complex I. *Sci Adv* **2023,** *9* (31), 1359). Similarly, more water is predicted for the central axis channel in L/ND5 subunit, as shown in the text. The reason for that could be poor resolution, or dynamic nature of molecules, or partial drying out of the samples during preparation; in particular, the latter seems likely in the E-channel connected to a big Q-cavity that could provide the channel of escape of hydrated water under drying conditions.

6. Insertion/Removal energies from protein water clusters (E-channel).

When there are clusters of hydrating water molecules, such as in the E-channel, to evaluate thermodynamic stability of the cluster the exact free energy of removal or insertion of individual water molecules needs to be calculated (and compared with the absolute chemical potential of bulk water). When one molecule is removed the whole cluster is affected due to water-water interaction within the cluster. Thus one needs to calculate the average energy of N and (N-1) water clusters and take their difference (removal or insertion energy). But this involves uncertainty due to thermal fluctuations of energies. Fig. S9 gives an estimate of energy distribution and uncertainty in such calculations.

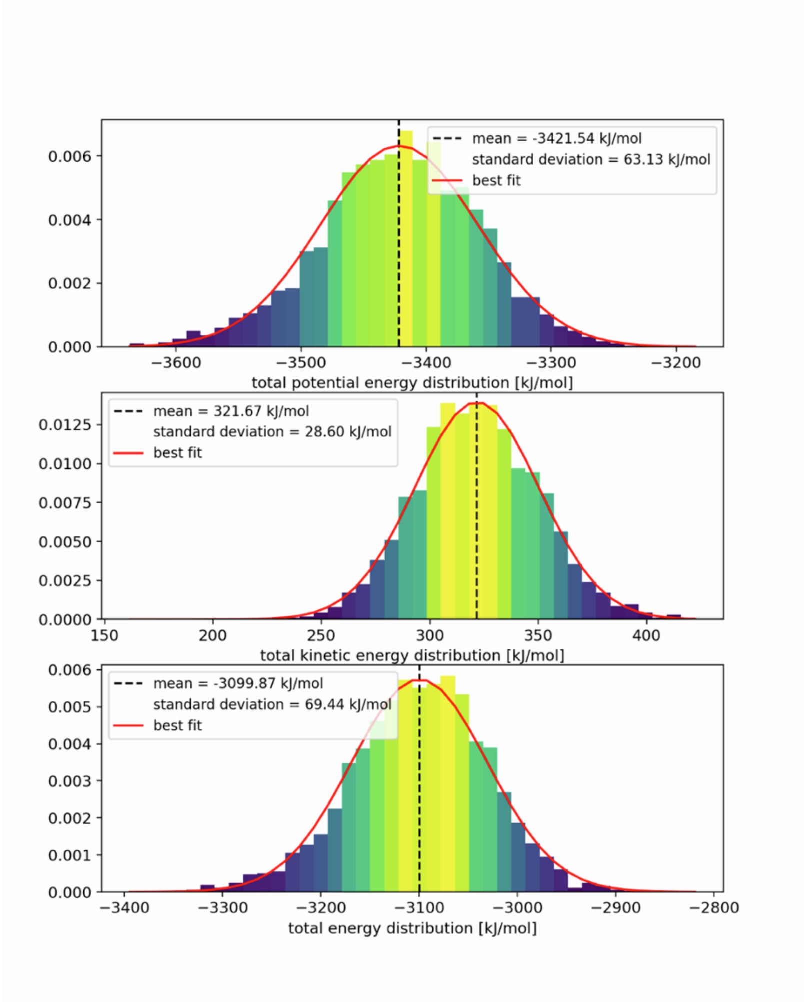

Figure S9. Energy (Potential, Kinetic, and Total) distribution of MD-simulated 42 Dowser++ water molecules in the *charged* structure of *Y. Lipolytica* extended E-channel. These and similar distributions were used to estimate the uncertainty (error bars) of the average energy of the clusters and resulting binding energies.

7. Effect of mutation in the gap region of E-channel (TM3/J/ND6).

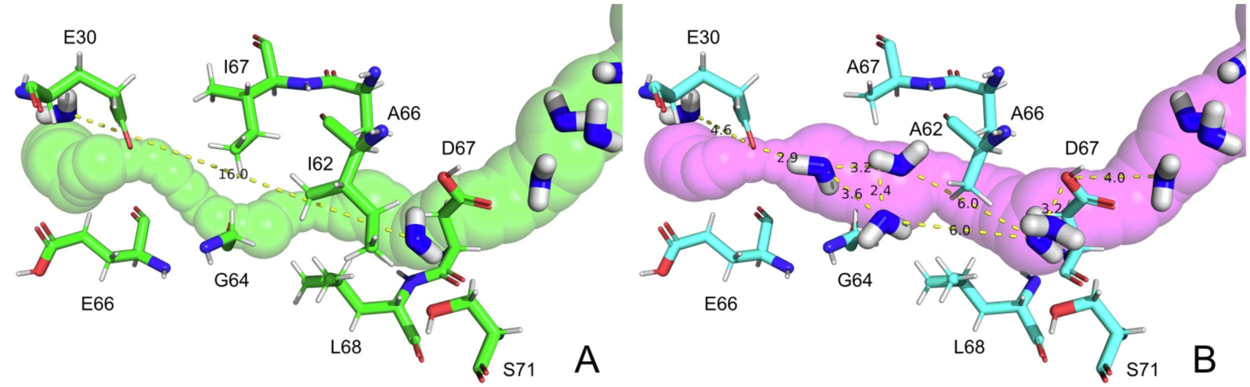

Figure S10. Shown in (A) is the channel gap between subunits A and K calculated using the *neutral* structure of *Y. lipolytica*. (The charged structure is shown in Fig. 12 in the main text.) The channel in (B) is calculated after mutating ILE 62 and ILE 67 into ALA 62 and 67. The Dowser++ predicted water molecules are shown as blue sticks. The nearby residues are shown as green sticks for the original structure and cyan sticks for the mutated structure. The 16.0 Å channel gap calculated for the neutral structure is shown in (A). Three additional water molecules were placed by Dowser++ due to the increased volume of the channel. These new water molecules broke the 16.0 Å gap into two smaller gaps of 4.6 Å and 6.0 Å, shown in (B). Notice that the same gap in charged structure, indicated by red arrows in Figure 12 (B), increased from 4.6 Å to 6.0 Å in neutral structure.

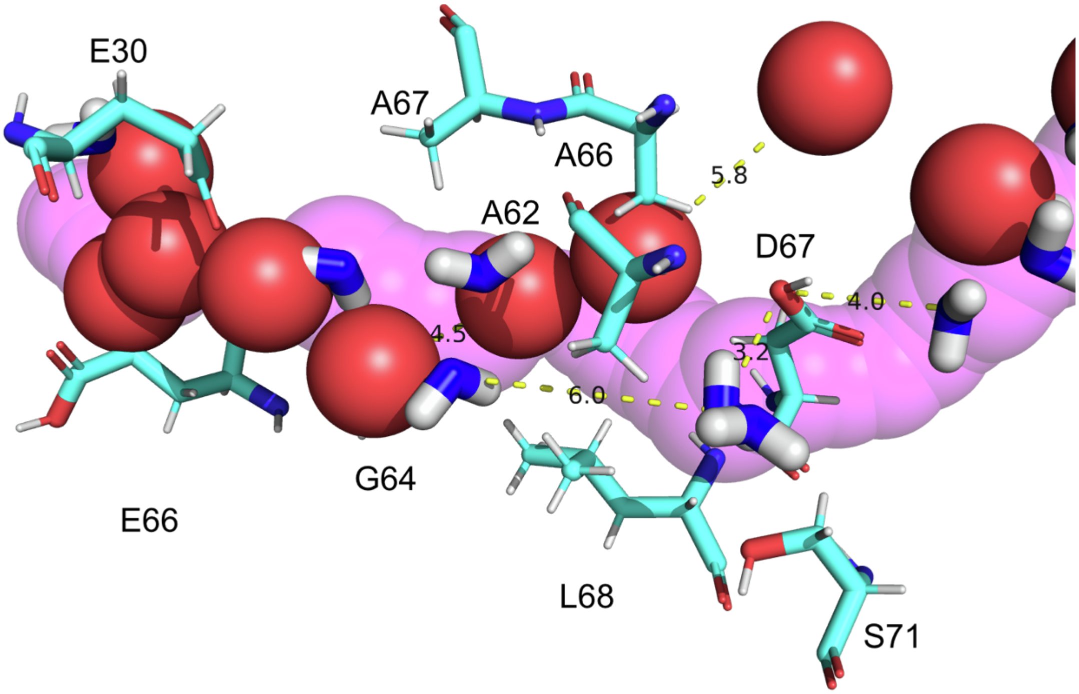

Figure S11. The channel between subunits A and K is calculated using the neutral structure of *Y. lipolytica*, after mutating ILE 62 and ILE 67 into ALA 62 and 67. Experimental waters from a newer mammalian structure [17] [1] (PDB code: 8OM1) are overlaid on top of *Y. lipolytica*

structure, shown as red spheres. The Dowser++ predicted water molecules are shown as blue sticks. The nearby residues are shown as cyan sticks. Three additional water molecules were placed by Dowser++ due to the increased volume of the channel. Two out of those three water molecules coincide with experimental waters. In addition, our prediction shows similar gaps of 6.0 and 4.0 Å long, comparing to 4.5 and 5.8 Å in the experiment. This 5.8 Å gap may be reduced by a nearby aspartic acid (D66 in 8OM1, D67 in 7O71 [2]) to 4.4 Å, as shown in [1].

[1] Grba, D.N., et al., *Investigation of hydrated channels and proton pathways in a high-resolution cryo-EM structure of mammalian complex I.* Sci Adv, 2023. **9**(31): p. eadi1359.
[2] Parey, K., et al., *High-resolution structure and dynamics of mitochondrial complex I-Insights into the proton pumping mechanism.* Sci Adv, 2021. **7**(46): p. eabj3221.
